# Feasibility of integrated, multilevel care for cardiovascular diseases (CVD) and HIV in low- and middle-income countries (LMICs): A scoping review

**DOI:** 10.1371/journal.pone.0212296

**Published:** 2019-02-22

**Authors:** Temitope Ojo, Lynette Lester, Juliet Iwelunmor, Joyce Gyamfi, Chisom Obiezu-Umeh, Deborah Onakomaiya, Angela Aifah, Shreya Nagendra, Jumoke Opeyemi, Mofetoluwa Oluwasanmi, Milena Dalton, Ucheoma Nwaozuru, Dorice Vieira, Gbenga Ogedegbe, Bernadette Boden-Albala

**Affiliations:** 1 Department of Epidemiology, New York University College of Global Public Health, New York, New York, United States of America; 2 New York University School of Medicine, New York, New York, United States of America; 3 Department of Behavioral Sciences and Health Education, College for Public Health & Social Justice, Saint Louis University, Saint Louis, Missouri, United States of America; 4 Section for Global Health, Department of Population Health, New York University School of Medicine, New York, New York, United States of America; 5 New York University Health Sciences Library, New York, New York, United States of America; 6 Department of Epidemiology, New York University College of Dentistry, New York, New York, United States of America; 7 Department of Neurology, Langone School of Medicine, New York University, New York, New York, United States of America; Purdue University, UNITED STATES

## Abstract

**Background:**

Integrated cardiovascular disease (CVD) and HIV (CVD-HIV) care interventions are being adopted to tackle the growing burden of noncommunicable diseases (NCDs) in low-and middle-income countries (LMICs) but there is a paucity of studies on the feasibility of these interventions in LMICs. This scoping review aims to present evidence of the feasibility of integrated CVD-HIV care in LMICs, and the alignment of feasibility reporting in LMICs with the existing implementation science methodology.

**Methods:**

A systematic search of published articles including systematic and narrative reviews that reported on integrated CVD-HIV care was conducted, using multiple search engines including PubMed/Medline, Global Health, and Web of Science. We examined the articles for evidence of feasibility reporting. Adopting the definition of Proctor and colleagues (2011), feasibility was defined as the extent to which an intervention was plausible in a given agency or setting. Evidence from the articles was synthesized by level of integration, the chronic care continuum, and stages of intervention development.

**Results:**

Twenty studies, reported in 18 articles and 3 conferences abstracts, reported on feasibility of integrated CVD-HIV care interventions. These studies were conducted in Sub-Saharan Africa, Southeast Asia and South America. Four of these studies were conducted as feasibility studies. Eighty percent of the studies reported feasibility, using descriptive sentences that included words synonymous with feasibility terminologies in existing definition recommended by Proctor and colleagues. There was also an overlap in the use of descriptive phrases for feasibility amongst the selected studies.

**Conclusions:**

Integrating CVD and HIV care is feasible in LMICs, although methodology for reporting feasibility is inconsistent. Assessing feasibility based on settings and integration goals will provide a unique perspective of the implementation landscape in LMICs. There is a need for consistency in measures in order to accurately assess the feasibility of integrated CVD-HIV care in LMICs.

## Introduction

Low- and middle-income countries (LMICs) continue to experience a significant double burden of chronic/non-communicable diseases (NCDs) and infectious diseases [1]. In order to address this public health challenge, several global and national efforts now embrace the integration of care for NCDs and chronic conditions, with infectious origins as a pragmatic strategy [1]. A prominent integrated model of care is that of cardiovascular diseases (CVD) and HIV/AIDs [1]. This model of integrated care was preceded by the aggressive global scale-up of care for HIV, especially in LMICs, which have the highest prevalence of HIV [1]. This scale-up for ART makes it possible for people living with HIV (PLHIV) to live longer, but also to develop comorbid chronic conditions including CVD and those that are CVD-related [1].

In line with implementation science frameworks, integrated CVD and HIV (CVD-HIV) evidence-based interventions (EBIs) should strive for maximum reach, efficacy, adoption, implementation, and maintenance, with an eventual goal of translating research into practice [2, 3]. This goal can only be attained if these interventions are feasible, within the settings where they are implemented [4]. Recent reviews of integrated CVD-HIV care in LMICs have focused on a combination of mostly clinical outcomes (CD4 counts, blood pressure, and HbA1c) and quality of care outcomes (improved quality of care) with little focus on implementation outcomes, particularly feasibility [4, 5, 6].

Implementation outcomes are important precursors to the long-term sustainability of EBIs [4]. To ensure the success of an integrated care design in reducing the chronic disease burden among individuals in LMICs, a critical analysis of early implementation stage outcomes for integrated CVD-HIV care is essential. Implementation outcomes occur in four early stages: acceptability, adoption, appropriateness, and feasibility [4]. According to Proctor, E. et al., acceptability *“is the perception among implementation stakeholders that a given treatment, service, practice, or innovation is agreeable, palatable, or satisfactory”[4].* Adoption *“is defined as the intention, initial decision, or action to try or employ an innovation or evidence-based practice”[4].* Appropriateness *“is the perceived fit, relevance, or compatibility of the innovation or evidence-based practice for a given practice setting, provider, or consumer; and/or perceived fit of the innovation to address a particular issue or problem” [4].* Feasibility *“is the extent to which a new treatment or innovation can be successfully used or carried out within a given agency or setting” [4]*. Other terminologies for feasibility include *actual fit* or *utility*, *suitability for everyday use* and *practicability [4]*. When purposively measured, these four implementation science outcomes are most salient during the early stages of the implementation process. Assessing the acceptability of an intervention by engaging stakeholders who are knowledgeable of the context is likely to facilitate the adoption of that intervention. Evaluating the appropriateness of the intervention for dealing with a problem and/or the setting, where the intervention is being introduced is also critical [4]. Feasibility further captures the successful or unsuccessful interrelationships between adoption, acceptability, and appropriateness [4]. Despite the importance of feasibility as an outcome for integrated CVD-HIV care EBIs, there is little data on rigorous assessment of this outcome in existing studies [5, 6].

Feasibility is integral to developing, evaluating and implementing complex EBIs such as integrated CVD-HIV care [7]. According to the Medical Research Council (MRC), complex interventions should be advanced in these four phases: development, feasibility and piloting, evaluation, and implementation [7]. Feasibility is assessed in the pilot phase and allows researchers/policy makers to anticipate early on, any uncertainty that may interfere with the eventual scale-up of the EBI [7, 8].

Additionally, implementation researchers are faced with the ongoing challenge of producing consistent taxonomy, definitions and measurements for monitoring implementation outcomes [4]. We used a scoping review to fully assess the feasibility of integrated HIV and CVD care in LMICs from the dearth of existing studies, given that a conventional systematic review approach would limit the scope of data to be retrieved based on its strict allowance of only RCTs [9]. This scoping review sought to answer the following question: Are multilevel integrated care of CVD and HIV conditions feasible in LMICs? The objectives of this scoping review on the feasibility of integrating CVD and HIV care in LMICs are to:

- Present the concept, taxonomy, and alignment of feasibility as reported in integrated CVD-HIV care studies in LMICs with established definitions in implementation science and intervention evaluation.
- Elucidate specific metrics used by researchers to assess the feasibility of integrated CVD-HIV care interventions in LMICs as a precursor to standardizing feasibility metrics, unique to implementation climate in LMICs.

## Methods

### Inclusion criteria

We included studies that reported on any component of an intervention used to integrate CVD management with HIV care, via quantitative, qualitative or mixed methods. According to the World Health Organization (WHO), integrated service delivery is *“the organization and management of health services so that people get the care they need*, *when they need it*, *in ways that are user-friendly*, *achieve the desired results and provide value for money”* [10]. For the purposes of this scoping review, integrated care for HIV and NCDs is defined as the strategic bringing together and operationalizing of services, delivery points, technologies, modified processes and management decisions occurring at different levels of service delivery, to manage HIV and CVD risk factors for patients with or without HIV/AIDS [10]. Integrated HIV and CVD care would be at facilities that originally offered only HIV services (Model 1), or only CVD services (Model 2), or neither HIV or CVD services (Model 3) [6], regardless of the type of care delivery setting (e.g. HIV clinics, primary care practices or community-based settings) [5, 6]. We focused on the most common CVD risk factors in patients with HIV and these include diabetes, hypertension, dyslipidemia (all of which are associated with metabolic syndrome—a common side effect of anti-retroviral drugs) [11]. Similarly, stroke was also included given its high association with hypertension [12]. We included studies that met the aforementioned criteria regardless of study design or whether or not the authors reported outcome measures. Studies were restricted to those reported in or translated to English. There were no date restrictions for the search. Included studies were restricted to those conducted in LMICs. LMICs was defined based on the World Bank classification of countries with Gross National Income per capita less than $995 to $12,055 in 2017 [13].

During the full-text review, feasibility was ascertained in selected articles if one or more of the following criteria were met: 1) a specific indication that a feasibility study was conducted; 2) descriptions of interventions with adjectives such as ‘feasible’ or terms synonymous with feasibility (actual fit or utility, suitability for everyday use and practicability); [4, 7] and 3) reporting one or more of the following early-stage implementation outcomes: acceptability, appropriateness, and adoption that contributes to the feasibility of EBIs [4].

### Exclusion criteria

Systematic reviews and literature reviews were excluded but were referenced in the background and discussion sections of this review. We excluded studies and reports that presented plans and recommendations for integrating CVD and HIV care without reporting on the actual implementation of these recommendations.

### Information sources

We conducted searches in the following databases: PubMed/Medline, Global Health, PubMed Central (PMC), Embase, Web of Science, Scientific Electronic Library Online (SCIELO), Food Science and Technology Abstracts (FSTA), Information Services for Physics, Engineering and Computing (INSPEC). Grey literature searches were conducted in Google Scholar, ResearchGate, the New York Academy of Medicine (New York AM) Grey Literature database, and in recently published systematic reviews on integrated chronic disease care.

### Search strategy

The Arksey and O’Malley framework for conducting scoping reviews guided the search for, identification of data; data extraction and data synthesis from selected studies [9](see **S1 Table**). An information specialist consulted on the search strategy design for different databases and sources. Databases were searched from their date of inception to April 13, 2018. Grey literature searches were concluded on May 18, 2018. The search strategy included the following terms and medical subject headings: healthcare settings, implementation science outcomes, hypertension, HIV infections and LMICs (see **S2 Table**). The Boolean logic strategy using a variation of keywords with the (AND/OR logic) was applied across all the databases.

### Study selection

Two authors (TO and LL) separately reviewed and assessed articles by article title or title and abstract, to determine articles met the inclusion criteria. A full-text review was then carried out to confirm that all articles selected met the inclusion criteria. Ambiguous abstracts were also evaluated via a full text review for eligibility. A third reviewer (SN) resolved disagreements between reviewers on an article’s eligibility (see **S1 Text**).

### Risk of bias assessment

The two reviewers assessed risk of bias independently. The Cochrane Risk of Bias Tool was used to assess risk of bias for RCTs [14]. This tool assessed each item of bias either as low, high or unclear risk of bias. The Newcastle-Ottawa Quality Assessment Scale was used to assess risk of selection bias, information bias and bias of confounding in non-RCT, observational studies [15]. Each item in this tool had multiple options, the lowest risk of bias being the option(s) with a star. Based on the guidance provided for determining risk of bias by both assessment tools, we assessed risk of bias in these three categories for all the studies: low risk of bias, high risk of bias and unclear risk of bias. Low risk of bias indicated that the item on the risk of bias assessment tool was described and well accounted for in the study, using the tool’s specifications for determination. High risk of bias indicated the item of bias was not sufficiently described and tackled in the study. Unclear risk of bias indicated that there was no information provided in the studies to determine if the specific item of bias was addressed in the studies.

### Data collection process

The two reviewers (TO and LL) used a standardized Google form to extract study characteristics and results from the full text article review. TO and LL resolved data discrepancies by consensus or by a third reviewer (SN). Final articles were chosen by consensus.

### Data items and synthesis

The following data items were collected on the selected studies: location of intervention/programming, duration of intervention or programming, type of healthcare setting, number of facilities/sites receiving the intervention or programming, if the location was rural, urban, peri-urban or both, the model of integration used, the target recipients of the intervention or programming, the types of staff used, if the staff were new, existing or a hybrid of both, context on reported feasibility of intervention, clinical outcomes, implementation outcomes and reviewers’ (TO and LL) notes from each article.

Due to the heterogeneity in the types of integrated care interventions, study design and reported outcomes, we conducted a qualitative synthesis of the scoping review, reporting data on feasibility as observed in selected studies.

Selected studies were reported by the: i) level of integration ii) stage of intervention development, iii) entry-point of intervention along the chronic disease continuum.

The three levels of integration were: micro-, meso-, and macro-levels of integrations [16]. Micro-level integration comprises of integrated CVD-HIV care interventions that are individual-level and patient-focused [16]. An example of micro-level integration of care would be coordinated care between professionals in charge of care for an individual patient, to ensure there is no break in communication and care continuum in the individual’s experience with the health system [16]. Meso-level integration represents delivery of integrated CVD-HIV care to a specific group of people with similar disease conditions [16]. An example of meso-level integration of care would be integrated care for the elderly population or people with specific, long-term conditions, as these populations have a higher use of care services that are most optimal when coordinated [16]. Macro-level integration represents the delivery of integrated CVD-HIV care on a larger, systems-level scale to a broader catchment of the population [16]. An example of a macro-level integration of care is Kaiser Permanente, one of the largest non-profit health maintenance organizations in the United States, which integrates health plan, providers who provide outpatient care, and hospitals or facilities that deliver inpatient care. Kaiser provides care for about 12.2 million people [16, 17]. At any of these levels, the integration could be clinical (shared guidelines and protocols for several clinical care processes) or service-oriented (multidisciplinary delivery of clinical services) [16]. Clinical integration involves fusing care provided by different providers and professionals for patients into a single process, navigated with use of shared guidelines and protocols [16]. Service integration occurs when different clinical services provided are integrated at an organizational level (hospital level or care-group level), aided with the use of multidisciplinary professional teams [16].

This review adopted the chronic disease continuum developed in the North West Adelaide Health Study, a large population-based cohort study investigating NCDs prevalence and related risk factors along the continuum [18]. Stages of chronic conditions were classified into those at risk of NCDs, those with a previously undiagnosed NCD, and those previously diagnosed with an NCD. The corresponding type of action for each of these stages in sequential order is: i) prevention, ii) delay/early detection, iii) prevention/ delay/early detection/ care [18]. Using the referent chronic disease continuum in this review, screening aligns with taking prevention and delay/early detection actions; referral/linkage to care aligns with taking delay/early detection actions and determination of care, if needed; and treatment of diagnosed conditions aligns with the taking prevention/delay or early detection/care actions.

The MRC recommended stages of intervention development used were: intervention design/development stage, the evaluation and implementation stage, and the intervention scale-up stage [7] (See **S1 Protocol**).

## Results

**Fig 1:**
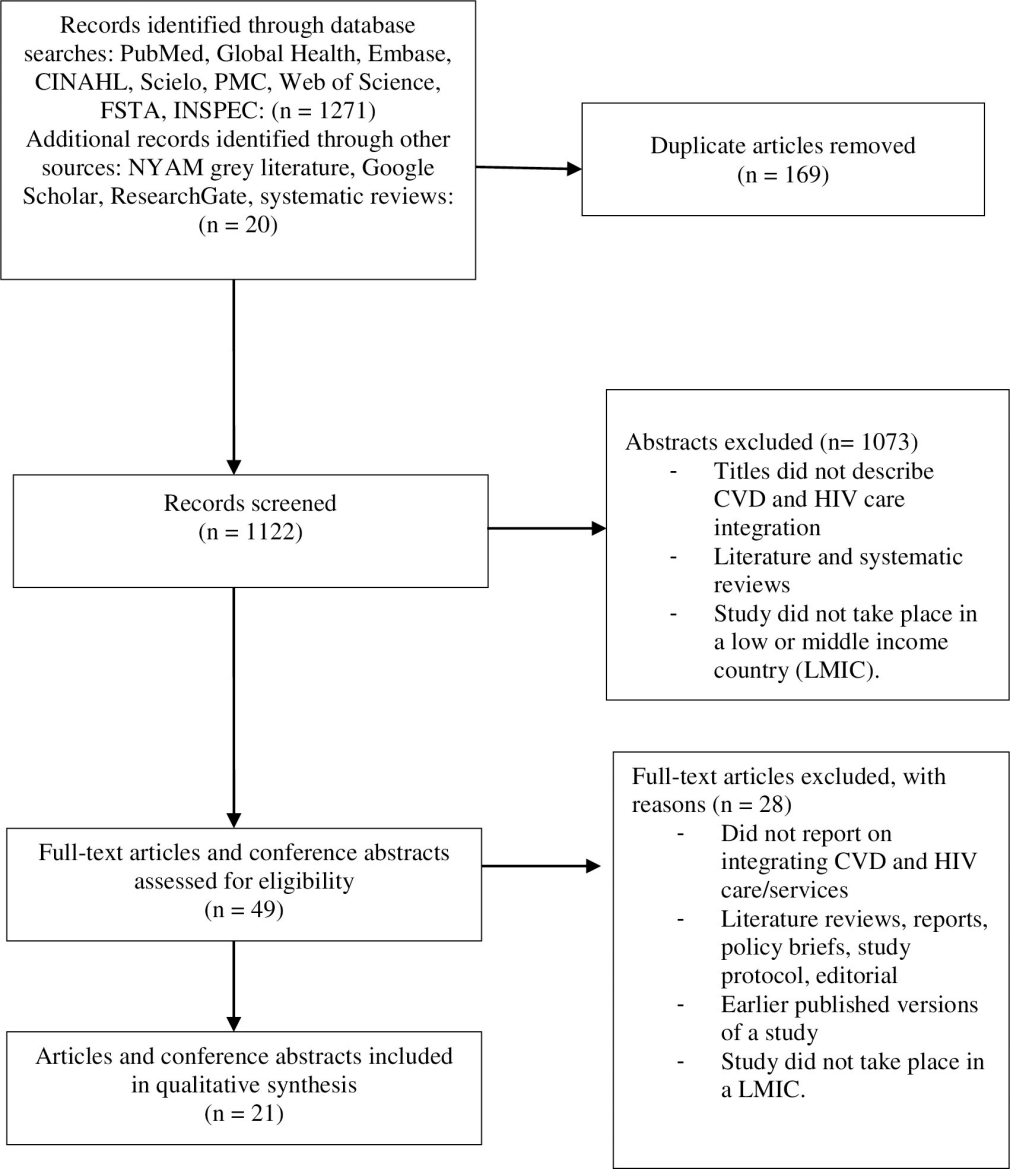
PRISMA flow diagram for paper appraisal.

A total of 1291 records were retrieved and 169 duplicate records were removed. Reviewers screened 1122 records by title and abstract, based on the inclusion criteria. Forty-nine articles met these inclusion criteria and were selected for full-text review. After full-text review, 28 of these articles were excluded. Data was extracted from a final selection of 18 papers and three conference abstracts that met the eligibility criteria for the review (See **Fig 1**).

Eighteen papers and three abstracts reported feasible integrated care interventions for 20 studies in 13 LMICs (See **Table 1**). Most of the selected studies were conducted in 11 countries in Sub-Saharan Africa (SSA) (n = 18). One study was conducted in Southeast Asia and another one in South America. Study duration ranged from one month to seven years. Both PLHIV and HIV-negative populations were targeted in 16 studies, while four studies only targeted PLHIV. Using models of integration as defined by Duffy, M. et al [6], 12 studies integrated CVD services into HIV services (Model 1 integration), two studies integrated HIV services into CVD services (Model 2 integration) and six studies integrated CVD and HIV services simultaneously (Model 3 integration). Additionally, only 5% of selected studies (n = 1) was a randomized controlled trial (RCT)—a pragmatic cluster RCT [19]. Most studies were either descriptive, cross sectional studies (n = 8) [20, 21, 22, 23, 24, 25, 26, 27] or observational cohort studies (n = 6) [21, 28, 29, 30, 31] (See **S1 Dataset**)

**Table 1:** Descriptive characteristics of final study selection.

Title (First Author, Year)	Duration of Study; Start year of intervention	Setting	†Model of Integration/ Level of integration	Target Population	Description of integrated components of intervention	Primary Outcomes and Findings
HIV with non-communicable diseases in primary care in Kibera, Nairobi, Kenya: characteristics and outcomes 2010–2013 (Edwards, et al., 2015) [20]	3 years, 5 months; 2010	Urban Kenya	Model 1/Meso level	*PLHIV and HIV negative	Lessons from ART provision, including treatment literacy, access to free medications and care, were applied with the integration of NCD and HIV/TB care in primary care clinics in Kibera.	Outcome: Statistical differences in blood pressure, HbA1c, fasting glucose and cholesterol levels between PLHIV and HIV negative patients Finding: Blood pressure (BP), HbA1c, fasting glucose and cholesterol levels did not significantly differ between PLHIV and HIV groups
Educational Outreach with an Integrated Clinical Tool for Nurse-Led Non-communicable Chronic Disease Management in Primary Care in South Africa: A Pragmatic Cluster Randomised Controlled Trial (Fairall, et al., 2016) [19]	21 months; 2011	Rural and Urban South Africa	Model 1/ Meso level	PLHIV and HIV negative	Primary Care 101 (PC101) is designed to support and expand nurses' role in NCD care, comprising educational outreach to nurses and a clinical management tool with enhanced prescribing provisions.	Outcome: Treatment intensification (increase in dose or number of medications or change in medication class). Finding: No significant improvements in treatment intensification for hypertension, diabetes, CKD, or case detection of depression between nurse managed and control clinics
Offering integrated care for HIV/AIDS, diabetes and hypertension within chronic disease clinics in Cambodia (Janssens, et al., 2007) [21]	5 years, 9 months; 2002	Rural Cambodia	Model 3/Meso level	PLHIV and HIV negative	Outpatient consultations, with services actively promoted as clinics for treatment of diabetes, hypertension and HIV/AIDS. Complementary services included counseling, provision of information and support on medication adherence and lifestyle changes.	Outcome: Progression of treatment (mortality and proportion still following up on treatment in clinics at 24 months). Finding: Patients alive and in active follow-up at 24 months: 87.7% (PLHIV); 71% (diabetics)
Novel approaches to screening for noncommunicable diseases: Lessons from Neno, Malawi (Kachimanga, et al., 2017) [22]	18 months; 2015	Rural Malawi	Model 1/ Meso level	PLHIV and HIV negative	Multi-disease screening programs that target nutritional disorders, hypertension, diabetes, HIV, tuberculosis (TB), and cervical cancer.	Outcome: Proportion of positive screening for hypertension and diabetes. Proportion referred for hypertension and diabetes care. Increase in patient NCD care enrollment due to screening intervention. Findings: Screened: 58% (for hypertension); 29% (for diabetes) Referred: 9% (for hypertension); 3% (for diabetes) Patients ever enrolled in NCD care every 3 months tripled 40 to 114.
Adaptation of a general primary care package for HIV-infected adults to an HIV center setting in Gaborone, Botswana (Davis, et al., 2013) [28]	1 year; 2012	Urban Botswana	Model 1/Macro level	PLHIV only	A package including screening for CVD, hypertension, hyperlipidemia and diabetes as well as other NCDs was adopted.	Outcome: An adapted Preventative care package with NCD recommendations Finding: An adapted Preventative care package with NCD recommendations
Family health days: an innovative approach to providing integrated health services for HIV and non- communicable diseases among adults and children in hard-to-reach areas of Lesotho (Tiam, et al., 2012) [32] [Abstract]	1 month; 2011	Rural Lesotho	Model 3/Meso level	PLHIV and HIV negative	Hypertension and diabetes screening were included w/ HIV screen from mobile clinics.	Outcome: Proportion of people who screened positive for HIV, hypertension and elevated blood sugar. Proportion of people linked to care. Findings: 68.5% of PLHIV received CD4 testing 36.6% of PLHIV were enrolled into HIV care 100% of HIV positive individuals linked to care were enrolled 24.4% of patients had hypertension and were linked to care 3.1% of patients had elevated blood sugar and were linked to care
Medication Adherence Clubs: a potential solution to managing large numbers of stable patients with multiple chronic diseases in informal settlements (Khabala, et al., 2015) [23]	12 months; 2013	Urban Kenya	Model 1/ Micro level	PLHIV and HIV negative	Medication Adherence Clubs (MACs) are nurse-facilitated mixed groups of 25–35 stable hypertension, diabetes mellitus and/or HIV patients who met quarterly to confirm their clinical stability, have brief health discussions and receive medication.	Outcomes: Percent provider compliance to hypertension, diabetes and HIV care protocol. Proportion of needed referral for clinical officer review; proportion lost to follow-up in MACs. Findings: 99% provider compliance to protocols 2% of patients referred back to clinic 3.5% Lost to follow up
Evaluating the feasibility and uptake of a community-led HIV testing and multi-disease health campaign in rural Uganda (Kabami, et al., 2017) [24]	5 months 3 weeks; 2014	Rural Uganda	Model 3/Meso level	PLHIV and HIV negative	Screening for HIV, hypertension, diabetes and malaria, male condom distribution, referral for medical circumcision for men, and family planning services for women in community-led Health campaign (CLHC).	Feasibility Outcomes: Elected leader acceptance and participation in intervention. Implementation of pre-campaign community mobilization activities. Implementation of pre-campaign census enumeration. Implementation of multi-disease screening services. Uptake outcomes: Proportion of community residents’ uptake of screening intervention. Measurement of community testing coverage. Screening outcomes: Proportion of residents screening positive for HIV, malaria, hypertension, and elevated blood sugar. Cost of intervention per participant. Findings: Feasibility: All leaders (N = 8) accepted and participated in the pre-campaign and campaign activities. All leaders designed and implemented community mobilization activities. All leaders implemented door-to-door census, enumerating 5,202 residents. Selected local clinic staff participated actively in CLHC. Ministry of Health made screening provisions available for the campaign. Uptake: 53% of residents participated. 93% HIV testing uptake by adult participants. Successful linkage of CLHC participants’ record to census enumeration records. Accountability and measurement of all services delivered with logbooks. Screening: 7.1% adults screened positive for HIV 27% screened for malaria; 5.3% malaria + 18.7% screened positive for hypertension (systolic BP ≥140mmHg or diastolic BP ≥90mmHg) 2.8% had elevated blood glucose (≥11.1mmol/L) Cost: $8.57 /participant
Preparedness of HIV care and treatment clinics for the management of concomitant non-communicable diseases: a cross-sectional survey (Leung, et al., 2016) [25]	1 month; 2013	Urban Tanzania	Model 1/Meso level	PLHIV only	Assessment of facility resources available for NCD diagnosis and treatment in U.S. President’s Emergency Plan for AIDS Relief (PEPFAR)–supported HIV Care and Treatment Clinics (CTCs) in Dar es Salaam, Tanzania.	Outcomes: Available resources and services for NCD care at patient, provider and clinic levels. Findings: 43% of clinics reported treatment of NCDs (hypertension) 21% of clinics had protocol for NCD management 21% of clinics had a trained NCD healthcare worker 14% of clinics provided education on diabetes, 57% on tobacco cessation, 64% on weight management and 86% on alcohol abuse.
Leveraging HIV platforms to work toward comprehensive primary care in rural Malawi: the Integrated Chronic Care Clinic (Wroe, et al., 2015) [26]	4 months; 2015	Rural Malawi	Model 1/ Meso level	PLHIV and HIV negative	Integrated chronic care clinic that utilizes a robust HIV program as a platform for NCD screening and treatment. Provision of longitudinal care for patients with an array of chronic diseases including HIV and common NCDs, allowing for a single visit for all of a patient's conditions. Complete integration of staff, patient flow, patient identification, and data-management.	Outcome: Increase number of facilities able to deliver the full Essential Health Package from 2 to 13 facilities. Findings: Dissolution of ART clinics and formation of Integrated Chronic Care Clinics (IC^3^). Coverage of IC^3^: utilized by 6781 patients on ART Utilized by 721 patients with NCD (379 with hypertension, 76 with diabetes) 15.1% PLHIV were among NCD patients
Prevalence and Knowledge Assessment of HIV and Non-Communicable Disease Risk Factors among Formal Sector Employees in Namibia (Guariguata, et al., 2015) [27]	21 months; 2009	Urban Namibia	Model 3/Meso level	PLHIV and HIV negative	A medical screening was conducted for HIV, blood glucose and blood pressure with pre and post testing, counseling, and referrals.	Outcome: prevalence of elevated blood pressure, elevated blood glucose and HIV. Knowledge and self-perceived risk of employees with chronic conditions. Findings: 25.8% had hypertension 8.3% had an elevated random blood glucose 8.9% were PLHIV Majority of patients could not identify risk factors for hypertension, diabetes or HIV
A time-motion study of cardiovascular disease risk factor screening integrated into HIV clinic visits in Swaziland (Palma, et al., 2018) [29]	9 months; 2015	Urban Swaziland	Model 1/ Meso level	PLHIV only	HIV clinic staff received training on and administered CVD Risk Factors screening (CVDRF) to patients during routine “refill appointments”. This consisted of point-of-care testing for total cholesterol and HbA1c, systolic and diastolic BP measurements, a structured interview to assess current smoking and medication use, and WHO/ISH risk stratification to predict 10-year risk of a cardiovascular event.	Outcome: Difference in visit times due to HIV and CVD risk factor screening. Findings: Screening increased median visit time from 4 to 15 minutes Time spent on HIV care was unaffected by screening. All interviewed patients would recommend screening to others
Linkage to HIV, TB and non-communicable disease care from a mobile testing unit in Cape Town, South Africa (Govindasamy, et al., 2013) [30]	1 year, 8 months; 2010	Urban South Africa	Model 1/ Meso level	PLHIV and HIV negative	Mobile testing unit provided screening for HIV, TB symptoms, diabetes and hypertension; health talks, referral letters, antenatal and reproductive health services.	Outcome: % yield of newly diagnosed cases of HIV, hypertension, TB and diabetes. Proportion linked to care. Findings: % of new diagnoses among screened patients: 5.5 (HIV); 10.1 (TB); 0.8 (diabetes); 58.1 (hypertension) % linked to care: 51.3 (HIV); 56.7 (TB); 74.1 (diabetes); 50.0 (hypertension)
Pinotti, J.A., et al., Comprehensive health care for women in a public hospital in Sao Paulo, Brazil (Pinotti, et al., 2001) [31]	7 years; 1991	Urban Brazil	Model 3/ Meso level	PLHIV and HIV negative	A health team oversees the integration of diagnostic and therapeutic services with a series of surveillance and preventive measures for women. Programs are set up to diagnose, detect, and treat diseases that are highly prevalent, such as cancer, STD, AIDS, hypertension, diabetes, etc.	Outcomes: proportion of women diagnosed for hypertension, obesity and HIV. Findings: % of women > 45 years old diagnosed: 21.2 (hypertension); 3.7 (Obesity); 0.7 (HIV) % of women < 45 years old diagnosed: 7.2 (hypertension); 3.1 (Obesity); 0.9 (HIV)
Screening for diabetes and hypertension in a rural low income setting in western Kenya utilizing home-based and community-based strategies (Pastakia, et al., 2013) [33]	1 month, 2 days; 2010	Rural Kenya	Model 1/Meso level	PLHIV and HIV negative	Home based screening for hypertension and diabetes by HIV counselors, and community based testing by district hospital staff.	Outcomes: Differences in likelihood of screening positive for hypertension or diabetes between home based screening and community based screening. Findings: Participants in community based screening were twice as likely to screen positive for hypertension compared to home based screening (OR = 1.93, p = 0.06). Participants in home based screening were 3.5 times more likely to screen positive for a random blood glucose level ≥7mmol/L, compared to community based screening (OR = 3.51, p<0.01). Low rate of follow-up for both community and home based screening, with no difference in rates between the two strategies.
Strengthening Health Systems at Facility- Level: Feasibility of Integrating Antiretroviral Therapy into Primary Health Care Services in Lusaka, Zambia (Topp, et al., 2010) [34]	6 months; 2007	Urban Zambia	Model 2/ Meso level	PLHIV and HIV negative	Clinics delivered HIV care and testing along with measuring vital sign in an integrated clinic.	Outcomes: Rates of HIV case finding and referral to care. Median waiting and consultation time. Findings: % of patients accepting testing: 53 (clinic 1); 58 (clinic 2) % of PLHIV patients: 13 (clinic 1); 24 (clinic 2) % of patients enrolled in care: 42 (clinic 1); 58 (clinic 2) Median waiting times: Clinic 1: Increased by 36 minutes and 23 minutes for ART and outpatient department (OPD) patients. Clinic 2: increased by 47 minutes and 34 minutes for ART and OPD patients, respectively. Consultation times: Clinic 1: Increased by 55% for OPD patients and decreased by 1% for ART patients. Clinic 2: Increased by 110% for OPD patients and decreased by 23% for ART patients.
Cardiovascular disease risk factor profiles of HIV- positive clients: finding from a pilot program to integrate CVD screening into HIV services at a secondary health facility in Kano, North-western Nigeria (Gwarzo, et al., 2012) [35] [Abstract]	15 months; 2010	Urban Nigeria	Model 1/ Meso level	PLHIV only	Integrated routine screening of cardiovascular risk factors in an HIV clinic.	Outcome: proportion of HIV patients screened positive for CVD risk factors. Findings: 19.8% of those screened identified with at least 1 CVD risk factor.
You can treat my HIV—But can you treat my blood pressure? Availability of integrated HIV and non-communicable disease care in northern Malawi (Pfaff, et al., 2017) [36]	2 years 1 month; 2012	Peri—urban Malawi	Model 1/ Meso level	PLHIV and HIV negative	Integration was in the form of both ART and NCD care administered in the same consultation, or administered in the same day but different consultation or both services were available in the same center but different days.	Outcome: capacity of ART sites to administer care for hypertension and diabetes. Findings: 60% of hospitals had 1 physician and 1 nurse trained in NCD care 5% of health centers had 1 physician trained in NCD care and 8% had 1 nurse trained in NCD care 48% of health centers provided ART and NCD care in the same consultation 1 hospital and no health centers screened for hypertension among ART patients
Evaluation of a project integrating cardiovascular care into HIV programmes (Nyabera, et al., 2011) [37] [Abstract]	16 months; 2009	Urban Kenya	Model 1/Meso level	PLHIV and HIV negative	Integration of CVD risk factor evaluation and management into HIV clinic settings.	Outcome: Monitoring and evaluation results of intervention. Findings: % of patients with hypertension: 19 (HIV-); 32 (PLHIV) % of ART patients with other NCDs: 6 (elevated blood glucose); 2 (hyperlipidemia) Staffing and equipment remained barriers to care
Effectiveness of an Integrated Approach to HIV and Hypertension Care in Rural South Africa: Controlled Interrupted Time-Series Analysis (Ameh, et al., 2017) [38] Quality of integrated chronic disease care in rural South Africa: user and provider perspectives (Ameh, et al., 2017) [39]	30 months; 2011	Rural South Africa	Model 3/ Meso level	PLHIV and HIV negative	The ICDM (integrated chronic disease management) model aims to improve health outcomes for patients being managed for HIV/AIDS, TB, hypertension, diabetes, chronic obstructive pulmonary disease, asthma, epilepsy, and mental health illnesses in PHC facilities.	Outcome: Effectiveness of integrated chronic disease management (ICDM) model in controlling patients’ CD4 counts (>350cells/mm^3^) and blood pressure (<140/90mmHg). Emerging themes from patient and provider narratives on ICDM model. Findings: Pilot facilities were 6% more likely to control CD4 counts and 1% more likely to control BP than comparison facilities Integration led to de-stigmatization for PLHIV Medication and equipment shortages limited treatment benefits for hypertension patients

*PLHIV- People living with HIV

^†^Model 1: NCD services are integrated into centers originally providing HIV care, programs started as HIV clinics and evolved to integrate screening, care and/or treatment of NCDs [6].

Model 2: HIV care is integrated into existing NCD care at primary healthcare delivery sites where patients receiving NCD care were also provided HIV testing and counseling and if screening is positive, HIV care and treatment [6]. Model 3: NCD and HIV care and treatment are simultaneously introduced and during outreach or at the same clinic site [6].

**Fig 2:**
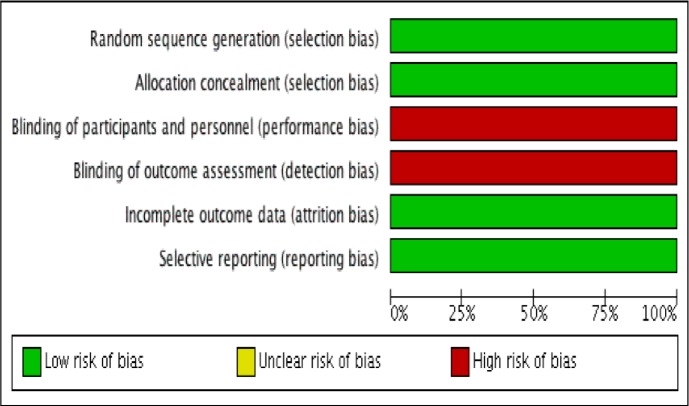
Risk of bias summary graph for RCT study. Risk of bias graph: review authors' judgments about each risk of bias item presented as percentages across all included RCT studies (n = 1). Source: *Review Manager (RevMan) [Computer program]*. *Version 5*.*3*. *Copenhagen*: *The Nordic Cochrane Centre*, *The Cochrane Collaboration*, *2014*.

The Cochrane risk-of-bias tool was used to assess the one RCT (See **Fig 2**). Random sequence generation, allocation concealment, incomplete outcome data and selective reporting were considered collectively as selection bias, which yielded a low risk of bias. There was a high risk of information bias due to inadequate blinding throughout the study. The authors of this study were unable to blind at the clinic level due to the nature of the intervention [19].

**Fig 3:**
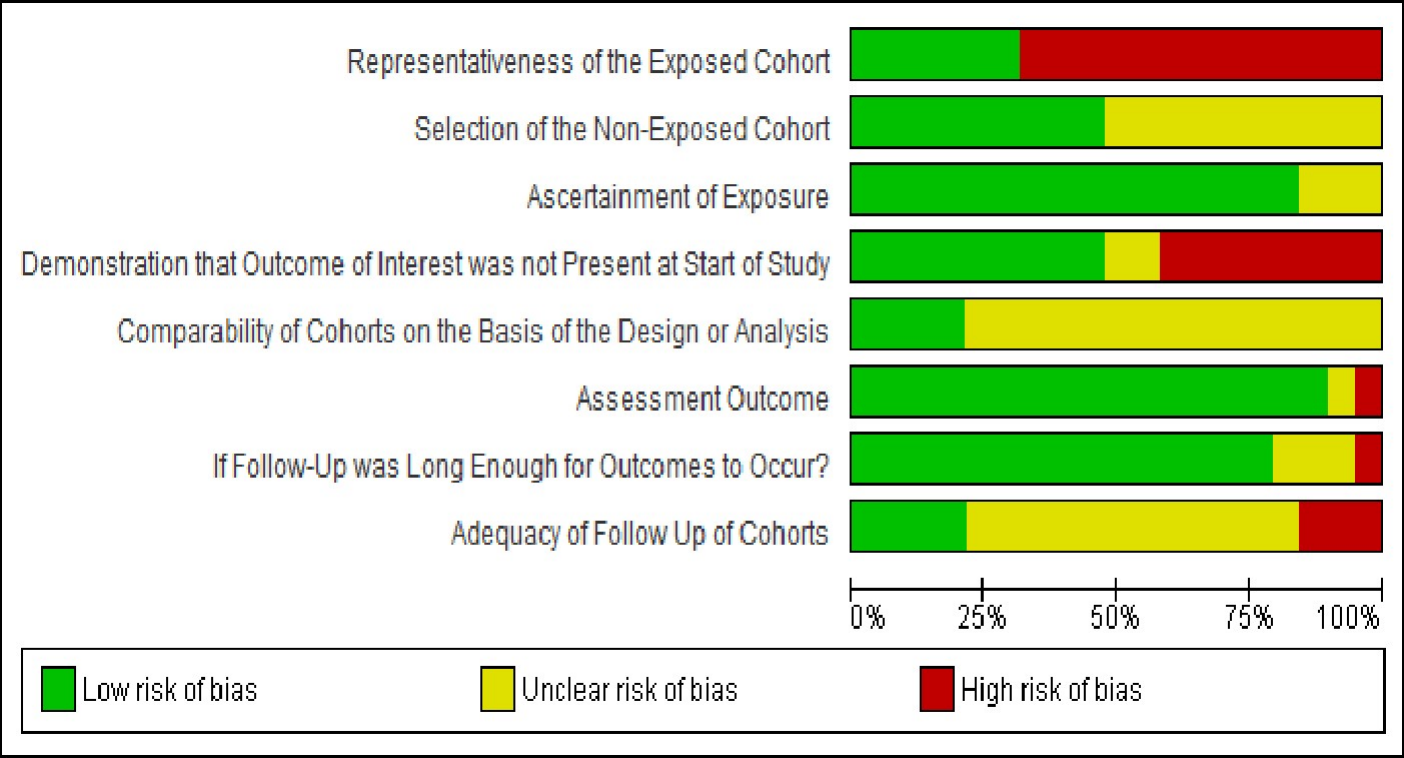
Risk of bias summary graph non-RCT studies. Risk of bias graph: review authors' judgments about each risk of bias item presented as percentages across all included non-RCT studies (n = 19). Source: *Review Manager (RevMan) [Computer program]*. *Version 5*.*3*. *Copenhagen*: *The Nordic Cochrane Centre*, *The Cochrane Collaboration*, *2014*.

The remaining non-RCT studies were assessed using the Newcastle-Ottawa Quality Assessment Scale and given a rating of high, low or unclear in terms of selection, information and confounding bias (See **Fig 3**). Selection bias was assessed based on representativeness of the exposed cohort, selection of the non-exposed cohort, length of follow-up time and adequacy of follow-up. Due to the distribution of studies across these categories, the risk of selection bias was unclear. Information bias was determined based on ascertainment of exposure and outcome; across the studies, there was a low risk of information bias. Based on the demonstration that the outcome of interest was not present at the start of the study and the comparability of cohorts in each study, the risk of bias due to confounding was unclear across the studies.

Of the 20 studies that were included after full-text review, only four studies conducted feasibility studies of an integrated CVD and HIV care intervention (See **Table 2**). Most of the studies (n = 16) reported on feasibility of integrated CVD-HIV care interventions in summary text with statements such as: the intervention ‘is feasible…’ [19, 20, 21, 22] ‘…can be utilized’ [28], and ‘ … can increase healthcare access’ [32] (See **Table 3**).

**Table 2:** Specific feasibility metrics used in selected studies.

Studies	Feasibility metrics used
Evaluating the feasibility and uptake of a community-led HIV testing and multi-disease health campaign in rural Uganda (Kabami, et al., 2017)	• Elected leader acceptance & participation in training for pre-campaign and campaign activities; • Implementation of pre-campaign community mobilization activities; • Implementation of pre-campaign census enumeration; • Implementation of health screening services, including point-of-care (POC) HIV, hypertension, diabetes and malaria screening; • Participation in community-led health campaign (CLHC) health services; • Measurement of uptake of health screening services, and community testing coverage.
Screening for diabetes and hypertension in a rural low income setting in western Kenya utilizing home-based and community-based strategies (Pastakia, et al., 2013)	• Measuring and comparing the proportion and likelihood of positive case detections for diabetes and hypertension in home-based and community-based screening; • Measuring and comparing the likelihood of follow-up after referral to manage a positive case detection of the two integrated screening strategies.
Strengthening Health Systems at Facility- Level: Feasibility of Integrating Antiretroviral Therapy into Primary Health Care Services in Lusaka, Zambia (Topp, et al., 2010)	• HIV case finding and referral rates; • Adherence to 6 clinical protocol indicators for patients on ART; • Median waiting time and consultation time; Patient and provider perceptions of integrated services.
Cardiovascular disease risk factor profiles of HIV- positive clients: finding from a pilot program to integrate CVD screening into HIV services at a secondary health facility in Kano, North-western Nigeria (Gwarzo, et al., 2012)	• Identification of CVD risk factors and • Determination of CVD risk percentile.

**Table 3:** Feasibility taxonomy and terminologies used in selected studies.

Studies	Feasibility terminologies used
Educational Outreach with an Integrated Clinical Tool for Nurse-Led Non-communicable Chronic Disease Management in Primary Care in South Africa: A Pragmatic Cluster Randomised Controlled Trial. (Fairall et al. 2016) [19] HIV with non-communicable diseases in primary care in Kibera, Nairobi, Kenya: characteristics and outcomes 2010–2013. (Edwards et al. 2015) [20] Offering integrated care for HIV/AIDS, diabetes and hypertension within chronic disease clinics in Cambodia. (Janssens et al. 2007) [21] Novel approaches to screening for noncommunicable diseases: Lessons from Neno, Malawi. (Kachimanga et al. 2017) [22]	The intervention ‘is feasible…’
Adaptation of a general primary care package for HIV-infected adults to an HIV centre setting in Gaborone, Botswana. (Davis et al. 2013) [28]	The intervention ‘can be utilized’
Family health days: An innovative approach to providing integrated health services for HIV and non-communicable diseases among adults and children in hard-to-reach areas of Lesotho. (Tiam et al. 2012) [32]	This intervention ‘can increase healthcare access’
Preparedness of HIV care and treatment clinics for the management of concomitant non-communicable diseases: a cross-sectional survey. (Leung et al. 2016) [25]	This intervention ‘maybe successfully achieved’
Educational Outreach with an Integrated Clinical Tool for Nurse-Led Non-communicable Chronic Disease Management in Primary Care in South Africa: A Pragmatic Cluster Randomised Controlled Trial. (Fairall et al. 2016) [19]	This intervention ‘is practical and acceptable’
A time-motion study of cardiovascular disease risk factor screening integrated into HIV clinic visits in Swaziland. (Palma et al. 2018) [29]	This intervention ‘has encouraging results’
Adaptation of a general primary care package for HIV-infected adults to an HIV centre setting in Gaborone, Botswana. (Davis et al. 2013) [28]	This intervention ‘is locally relevant’
Linkage to HIV, TB and non-communicable disease care from a mobile testing unit in Cape Town, South Africa. (Govindasamy et al. 2013) [30]	This intervention ‘can be used effectively’
Evaluating the feasibility and uptake of a community-led HIV testing and multi-disease health campaign in rural Uganda. (Kabami et al. 2017) [24] Kotwani P, Kwarisiima D, Clark TD, Kabami J, Geng EH, Jain V, et al. Epidemiology and awareness of hypertension in a rural Ugandan community: a cross-sectional study. (Kotwani et al, 2013) [40]	This intervention ‘is complementary and efficient’
HIV with non-communicable diseases in primary care in Kibera, Nairobi, Kenya: characteristics and outcomes 2010–2013. (Edwards et al. 2015) [20]	This intervention ‘is likely to benefit from NCD screening and treatment within similar HIV programs’
Quality of integrated chronic disease care in rural South Africa: user and provider perspectives. Health policy and planning. (Ameh et al. 2017) [39]	This intervention ‘demonstrated benefits’
Leveraging HIV platforms to work toward comprehensive primary care in rural Malawi: the Integrated Chronic Care Clinic. (Wroe et al. 2015) [26]	This intervention ‘improved accessibility’
Quality of integrated chronic disease care in rural South Africa: user and provider perspectives. (Ameh et al. 2017) [39]	This intervention ‘is yet to be achieved’
You can treat my HIV—But can you treat my blood pressure? Availability of integrated HIV and non-communicable disease care in northern Malawi. (Pfaff et al. 2017) [36] Evaluating the feasibility and uptake of a community-led HIV testing and multi-disease health campaign in rural Uganda. (Kabami et al. 2017) [24] Cost and Efficiency of a Hybrid Mobile Multidisease Testing Approach With High HIV Testing Coverage in East Africa. (Chang et al. 2016) [41]	This intervention ‘has much potential’
Pinotti JA, Tojal ML, Nisida AC, Pinotti M. Comprehensive health care for women in a public hospital in Sao Paulo, Brazil. (Pinotti et al 2001) [31]	Study concluded there was ‘feasibility of action strategy and economic feasibility’ in the
Medication Adherence Clubs: a potential solution to managing large numbers of stable patients with multiple chronic diseases in informal settlements. (Khabala et al. 2015) [23]	Study concluded there was ‘feasibility and early efficacy’

There was an overlap in terminologies (practicability and utility) used for feasibility between existing definitions of feasibility and those of selected studies [4]. Selected studies also reflected a trend of using terms for appropriateness (‘locally relevant’), acceptability (‘practical and acceptable), and adoption (the mere act of piloting interventions) to complement a conclusion that an intervention is feasible. Terminologies such as ‘demonstrated benefits’, ‘complementary and efficient’ align with the definition of feasibility as the extent to which an intervention can be used or implemented successfully in a specific setting (See **Table 3**) [4].

Four studies were conducted as feasibility studies; using specific metrics to assess feasibility (See **Table 2**). Some of the metrics used in these four studies overlap with the metrics recommended in publications such as acceptability, participant recruitment and retention rates (elected leaders’ acceptance and participation in training activities), compliance, and retention rates (likelihood of follow-up on a referral after positive case detection for diabetes or hypertension) [3, 4, 7, 8].

Outcome measures that contributed to a feasibility narrative across the studies include: population penetrance/participant recruitment rate, case detection, health knowledge, referral rates, new enrollment in care, and lost-to-follow up (LTFU) rates/participant retention rate/defaulter tracing (See **Table 4**). Predictors of feasibility were reported by several outcomes such as availability of resources and equipment, availability of staff, and reduced stigma (See **Table 4**). Of these outcome measures, case detection was the most frequently used (n = 13). Integrated interventions fell into three types: screening, referrals and linkage to care, and treatment. Multiple studies (n = 13) had more than one type of integrated intervention component.

**Table 4:** Outcomes by Intervention type.

Intervention	Outcome Reported	n (of studies) =
Screening		
	Population penetrance^1^	3
	Case detection^2^	13
	Health knowledge^3^	1
Referral/ Linkage to Care		
	Referral rates^4^	6
	New enrollment in care^5^	5
	LTFU rates/ Defaulter tracing^6^	6
Treatment		
	Disease management^7^	8
	Availability of resources and equipment^8^	5
	Availability of staff^9^	6
	Reduced stigma^10^	2

^1^ Reference numbers [22], [26], [38]

^2^ Reference numbers [22], [24], [25], [26], [27], [29], [30], [31], [32], [33], [34], [35], [37]

^3^ Reference numbers [27]

^4^ Reference numbers [22], [24], [30], [32], [33], [35]

^5^ Reference numbers [22], [26], [30], [32], [34]

^6^ Reference numbers [23], [30], [32], [33], [34], [39]

^7^ Reference numbers [19], [20], [21], [23], [25], [31], [33], [38]

^8^ Reference numbers [25], [26], [36], [37], [39]

^9^ Reference numbers [25], [26], [34], [36], [37], [39]

^10^ Reference numbers [34], [39]

Reporting of feasibility catered to the settings and/or scale-up of the interventions, helping identify unique facilitators and barriers to implementing integrated CVD-HIV care interventions (screening, referral/linkage to care and treatment) in LMIC settings. Barriers include lack of tracking of linkage to care after cases are referred (setting and scale-up specific) [22, 24, 29] loss to follow-up (setting-specific) [20], lacking time to make clinic visits during the weekdays (setting-specific) [30], using non-standard BP cutoffs (setting-specific) [33], insufficient funding (setting and scale-up specific) [28], cumbersome curriculum for training (scale-up specific) [19], and challenges in staffing and equipment (setting-specific) [37].

Facilitators include use of existing trained staff, training of healthcare personnel, use of local leaders (setting and scale-up specific) [22, 26, 28], free-of -charge care (setting-specific) [30], continuous medical supervision (setting-specific) [31], broad acceptability of intervention components (setting-specific) [29], having patient feedback (setting-specific) [29], enthusiastic buy-in, adoption, and acceptability from policy makers (scale-up specific) [19].

In this review, 18 studies reported meso-level integrated CVD and HIV interventions, seven of which were service integrations and 11 were clinical integrations; one study reported micro-level, clinically integrated intervention [23] and one study reported macro-level, clinically integrated CVD-HIV care intervention [28] (See **Table 5**).

**Table 5:** Prevalence of feasible studies by levels of integration.

Level of Integration	n (of studies) =
Micro Level	
Service	0
Clinical^11^	1
Meso Level	
Service^12^	7
Clinical^13^	11
Macro Level	
Service	0
Clinical^14^	1

^11^ Reference numbers [23]

^12^ Reference numbers [20], [24], [32], [34], [36], [37], [38], [39]

^13^ Reference numbers [19], [21], [22], [25], [26], [27], [28], [29], [30], [31], [32], [33], [35]

^14^ Reference numbers [28]

Most studies were in the evaluation and implementation stage of development (n = 18), while the scale-up stage of development had the least number of studies (n = 3). Studies (n = 2) overlapped in more than one stage of intervention, which confirms the iterative quality to stages of intervention development (See **Table 6**) [7]. Of the feasible studies, 16 studies had a multi-disease screening integrated component, 11 studies featured an integrated component with referral/linkage to care and 14 studies featured an integrated treatment component. By disease condition, hypertension had the most studies with an integrated multi-disease screening component (n = 16) and referral/linkage component (n = 10) while HIV encompassed the most studies with a treatment component in the intervention (n = 14). Diabetes had comparable number of studies as HIV with a screening component (n = 14) but had the lowest number of studies with a referral/linkage component (n = 9) and treatment component (n = 9) (See **Table 6**).

**Table 6:** Prevalence of feasible studies by chronic disease continuum targeted health actions.

Intervention type/ Corresponding stage of chronic condition^β^	Disease	Intervention Development * n (number of studies)	Evaluation and Implementation Work * n (number of studies)	Scale up * n (number of studies)	Total number of studies
Screening/risk of NCDs, undiagnosed NCD					16
	HIV	1 ^15^	13 ^16^	1^17^	
	TB	1 ^18^	3 ^19^	0	
	Hypertension	1 ^20^	15 ^21^	0	
	Diabetes	1 ^22^	13 ^23^	0	
Referral/ Linkage to Care/undiagnosed NCD, previously diagnosed NCD					11
	HIV	0	6 ^24^	0	
	TB	1 ^25^	2 ^26^	0	
	Hypertension	0	10 ^27^	0	
	Diabetes	1 ^28^	8 ^29^	0	
Treatment/previously diagnosed NCD					14
	HIV	1 ^30^	11 ^31^	2 ^32^	
	TB	0	0	1 ^33^	
	Hypertension	1 ^34^	8 ^35^	1 ^36^	
	Diabetes	0	8 ^37^	1 ^38^	

* The MRC recommended stages of intervention development

^β^ The North West Adelaide Health Study classified chronic disease continuum by stages of chronic conditions namely: those at risk of NCDs, those with a previously undiagnosed NCD, and those previously diagnosed with an NCD. The corresponding type of actions for each of these stages of disease in sequential order is: i) prevention, ii) delay/early detection, iii) prevention/ delay/early detection/ care. Using the referent chronic disease continuum, screening aligns with taking prevention and delay/early detection actions; referral/linkage to care aligns with taking delay/early detection actions and determination of care, if needed; and treatment of diagnosed conditions aligns with the taking prevention/delay or early detection/care actions.

^15^ Reference number [28]

^16^ Reference numbers [21], [22], [24–27], [29–33], [36], [37]

^17^ Reference number [24]

^18^ Reference number [28]

^19^ Reference numbers [22], [25], [30]

^20^ Reference number [28]

^21^ Reference numbers [21], [22], [24], [32], [37]

^22^ Reference number [28]

^23^ Reference numbers [21], [22], [24–27], [29–33], [36], [37]

^24^ Reference numbers [22], [24], [30], [32], [33], [36]

^25^ Reference number [28]

^26^ Reference numbers [22], [30]

^27^ Reference numbers [22], [24], [25], [29], [30], [32], [36]

^28^ Reference number [28]

^29^ Reference numbers [22], [24], [25], [29], [30], [32], [36]

^30^ Reference number [28]

^31^ Reference numbers [21], [23], [25], [26], [29], [31], [34], [37], [39]

^32^ Reference numbers [19], [20]

^33^ Reference number [19]

^34^ Reference number [28]

^35^ Reference numbers [20], [21], [23], [25], [31], [36], [37], [39]

^36^ Reference number [19]

^37^ Reference numbers [19], [20], [21], [23], [26], [31], [36], [37]

^38^ Reference number [19]

## Discussion

This scoping review is the first to collate reporting of the feasibility of integrated CVD-HIV care interventions in LMICs. Unlike recent literature reviews, which focused on the prevalence of integrated CVD-HIV care interventions and the models of integrations, this review targets the manner in which feasibility was assessed and reported in these interventions [5, 6].

Only four studies were designed as feasibility (mixed-methods) studies [24, 33, 34, 35]. Multi-disease screening was the most featured feasible integrated intervention in this review. This reflects a preferred prioritization in LMICs for prevention and early detection of chronic conditions. Referral and linkage to care as an integrated CVD-HIV care intervention was the least incorporated action across the selected studies (n = 11). In addition, of the 20 feasible integrated CVD-HIV care studies, 90% were implemented at the meso-level of integration, where groups of people with similar disease conditions were targeted for the intervention.

The results of the current review suggest that we have amassed increasing quantity of qualitative and descriptive data on the integration of CVD and HIV care in LMICs. This, to a large extent, suggests that the concept and reporting of feasibility in the 20 studies reviewed intersected with the definitions and terminologies for feasibility as an implementation and evaluation outcome. The metrics used to capture feasibility in these studies also align with some recommended metrics from Proctor and colleagues (2011), the MRC, amongst others, for assessing feasibility in complex interventions [3, 4, 7, 8]. This study provides additional understanding of the different components as feasibility in reviewed studies was reported as i) setting-specific viable intervention components, viable conditions under which intervention components work or would work better, (n = 17) [20, 21, 22, 23, 25, 26, 27, 28, 29, 30, 31, 33, 34, 35, 36, 37, 38] and ii) plans to move interventions into the scale-up phase or ongoing scale-up of interventions, due to proof of successful outcomes from pilot studies (n = 5) [19, 23, 24, 28, 32].

Although 80% (n = 16) of the studies reported and concluded that integrated CVD-HIV care interventions were feasible, the majority have no clear definitions of feasibility. This highlights the evident need for a consistent definition of feasibility in studies focused on assessing whether CVD and HIV care integration is viable. In addition to definitions, methodological rigor must be improved. Complex interventions such as those integrating CVD and HIV care are not easy to evaluate. We found limited use of tested feasibility metrics and methodology for assessing feasibility among researchers implementing integrated CVD-HIV care interventions in LMICs.

The use of a standard list of metrics for measuring feasibility would be an ideal means of promoting consistent methodology for assessing intervention feasibility [4, 7, 8] (See **Table 7**). Though there was an intersection between some recommended feasibility metrics from existing literature and the selected studies, there remain challenges in outlining a standard list of specific metrics that can accurately indicate EBI feasibility based on its definition as an implementation outcome [4, 7].

**Table 7:** Recommended list of feasibility indicators and their metrics for the three main types of intervention for HIV-CVD integrated care in LMICs.

Type of Intervention	Feasibility Indicator^‡^	Metric^‡^
Screening		
	Acceptability	Patient willingness to undergo screening. Likelihood that patient would recommend screening to a friend or family member. Community- wide promotion of screening efforts. Provider and stakeholders’ approval of screening protocols.
	Adoption	Population penetrance of screening effort. Population participation by demographic (sex, age, occupation, etc.). Creation of organization/setting polic(ies) to accommodate screening intervention.
	Appropriateness	Number of newly identified cases. False positive rate, specificity and sensitivity of screening intervention. Patient understanding of risk factors, screening results and next steps (health knowledge). Providers’ perception of screening intervention’s alignment with organization/setting and its mission. Cost/ Benefit analysis for organization/setting.
	Feasibility	Resource availability and training requirements to support a screening intervention.
Referral/ Linkage to Care		
	Acceptability	Patient compliance with enrollment instructions. Providers’ approval of enrollment, referral and linkage to care protocols.
	Adoption	Pre-post tests of: patients’ enrollment numbers in care, patients’ referral rates from providers, patients’ loss-to-follow-up defaulter tracing rates, providers’ enrollment, referral and linkage to care numbers. Creation of organization/setting polic(ies) to accommodate referral/linkage to care intervention.
	Appropriateness	Proximity/ availability of referral sites. Patient understanding of follow-up procedures and necessity. Patient and provider indicated barriers and facilitators to follow-up care. Providers’ perception of referral/linkage to care intervention’s alignment with organization/setting and its mission.
	Feasibility	Resource availability and training requirements to support referral/linkage to care intervention.
Treatment		
	Acceptability	Patient waiting times to receive service. Likelihood that patient would recommend treatment intervention to a friend or family member. Effects of treatment intervention on stigmatization of disease. Effects of treatment intervention on provider workload. Effects of treatment intervention on organization/setting capacity. Early signals of treatment intervention fit in organization/setting structure.
	Adoption	Patient engagement with and adherence to treatment plan. Provider use and adherence to established treatment protocol. Creation of organization/setting polic(ies) to accommodate treatment intervention.
	Appropriateness	Clinical outcomes of disease management. Patient understanding of treatment plan. Patient and provider indicated barriers and facilitators to treatment (resource availability, cost, staffing, etc.). Organization/Setting barriers and facilitators to treatment (facilities, cost, space, etc). Providers’ perception of treatment intervention’s alignment with organization/setting and its mission.
	Feasibility	Resource availability and training requirements to support treatment intervention.

^‡^ Researchers are encouraged to use quantitative and qualitative methods to measure feasibility metrics. It is suggested that measuring more of the indicators and metrics in the table will give a more complete assessment of an intervention’s feasibility.

There were only a few RCTs of integrated CVD-HIV care interventions in LMICs. Notably, complex interventions literature is shifting towards accepting, as evidence, non-RCT studies for interventions that cannot be pragmatically tested with RCT designs. Here, factors such as settings, resources, ethics, and urgency of intervention needs are prioritized over the gold-standard of research design [7]. Consequently, developing standard feasibility metrics will accommodate different research designs [7]. Overall, greater rigor is needed to provide the contextual information necessary to address the issues of case detection and referral among patients or across multiple sites tackling both CVD and HIV care. There is also a need for a change in methodological approaches. Although quantitative and qualitative data such as surveys, interviews, administrative reports are collected, the data represents a single time point. In order to collect data that will provide a better understanding on whether interventions are actually feasible, more longitudinal studies and longer periods of data collection are essential. This data would allow for a thorough feasibility assessment that facilitates moving an intervention forward for a full-scale evaluation and implementation, or going back to the design board to refine and improve certain intervention components [7].

Promoting training programs, specifically to teach implementation science methodology will address the evident challenges in methodological rigor for assessing feasibility of EBIs to integrate CVD and HIV care. A recent systematic review that explored implementation science outcomes in integrated NCD and HIV interventions in SSA, recommended implementation science capacity building as a means to guide the integration of NCD and HIV services in SSA [42]. Capacity building was modeled after ongoing training initiatives and institutional partnerships and spearheaded by institutions such as the National Institutes of Health (NIH) and the WHO [42].

At the national level, policymakers should accelerate the adoption of a policy tool, such as the WHO’s NCD Multisectoral Action Plan (MAP) tool, which has a provision for the implementation, monitoring, and evaluation of NCD prevention and control initiatives. With the NCD MAP tool, research on integrated EBIs for CVD and HIV can better align with nationally recognized gaps and priorities [43]. The NCD MAP tool allows for population-wide information gathering and sharing. This is useful for anticipating implementation challenges for EBIs integrating CVD-HIV care.

To improve research practice around measuring implementation outcomes like feasibility in LMICs, there should be a platform for researchers to share evidence-based measures, and reach a consensus on best practices. One such model is the web-based collaborative initiative, Grid Enabled Measures (GEM)–a NIH Implementation Science practice tool [44]. With GEM, researchers share and rate implementation measures and harmonize data on the use of these measures [44].

The data reported in this scoping review seeks to inform the evaluation of the implementation process in efforts to integrate CVD and HIV care for those aiming to do so in LMICs. This could enhance efficiency in the implementation of EBIs in LMICs, despite the limited resources available to address NCDs and HIV comorbidity in these countries [45, 46].

The breadth of this scoping review is wider than the most recent systematic and narrative reviews conducted on the integration of CVD-HIV services in LMICs [5, 6]. The scoping review concept and methodology allowed for a more conceptual investigation of terminologies and tone of data reporting, used by researchers to determine feasibility of integrated chronic disease care in LMICs [9]. With a scoping review approach, we presented a more comprehensive picture of feasibility assessment and reporting within implementation research in LMICs. A limitation of this review was that some selected studies were reported as conference abstracts with no published, full-length manuscripts to provide more context regarding the intervention [32, 35, 37].

## Conclusion

Several feasible interventions that integrated, multilevel CVD and HIV care were identified in this review. There is overwhelming evidence that assessment of feasibility was not conducted in a consistent fashion across studies, though all studies intersected, to varying degrees, with definitions and characterization of feasibility as an implementation or evaluation outcome. Selected studies used a reliable collection of additional metrics to inform on feasibility, besides recommended metrics. These metrics might inform the creation of a standard list of feasibility metrics, fitting for the implementation and evaluation landscape in LMICs. Most of the studies reported feasibility more for setting-specific purposes, at the evaluation and implementation stage of intervention development, but without a systematic method to measure feasibility. It is essential to adopt a rigorous and consistent methodology to report evidence of intervention feasibility, early in the implementation timeline, and prior to scaling up in LMICs.

## Supporting information

S1 Table Consolidated checklist for reporting scoping reviews (Arksey-O’Malley framework and PRISMA checklist). (DOCX)

S2 Table Search terms used to identify relevant studies. (DOCX)

S1 Text List of Abbreviations. (DOCX)

S1 Protocol PLOS scoping review protocol. (DOCX)

S1 Dataset Feasibility scoping review dataset. (XLSX)
