# Supplementary material for: Feasibility of integrated, multilevel care for cardiovascular diseases (CVD) and HIV in low- and middle-income countries (LMICs): A scoping review
Source: PLoS One. 2019 Feb 22;14(2):e0212296. doi: 10.1371/journal.pone.0212296 (PMC6386271; doi:10.1371/journal.pone.0212296)
Supplement: S2 Table — (DOCX) [file pone.0212296.s002.docx]

**S2 Table**

Search terms used to identify relevant studies

| Problem/Population | Concept 1  (developing countries OR developing country OR medically underserved area OR medically underserved areas OR lmic OR low income countries OR low income country OR middle income countries OR middle income country OR global OR resource poor OR low resource OR third world country OR third world countries OR africa OR central asia OR western asia OR southeastern asia OR indian ocean islands OR central america OR south america OR eastern europe OR transcaucasia OR china OR korea OR mongolia OR mexico OR caribbean region OR pacific islands OR africa OR central asia OR western asia OR southeastern asia OR indian ocean islands OR central america OR south america OR eastern europe OR transcaucasia OR caribbean OR pacific islands OR afghan OR afghan OR afghanistan OR bangladesh OR bangladeshi OR benin OR beninese OR burkina faso OR burkinabes OR burundi OR burundian OR cambodia OR cambodian OR central african republic OR central african OR chad OR chadian OR comoros OR comorian OR congo OR congolese OR eritrea OR eritrean OR ethiopia OR ethiopian OR gambia OR gambian OR guinea OR guinea OR haiti OR haitian OR kenya OR kenya OR korea OR korean OR kyrgyz OR kyrgyzstan OR liberia OR liberian OR madagascar OR malagasy OR malawi OR malawian OR mali OR malian OR mozambique OR mozambican OR myanmar OR myanmarese OR burmese OR nepal OR nepalese OR niger OR nigerian OR rwanda OR rwandan OR sierra leone OR sierra leoneans OR somalia OR somalia OR tajikistan OR tajik OR tadzhik OR tanzania OR tanzanian OR togo OR togolese OR uganda OR ugandan OR zimbabwe OR zimbabwean OR angola OR angola OR armenia OR armenian OR belize OR belizean OR bhutan OR bhutanese OR bolivia OR bolivian OR cameroon OR cameroonian OR cape verde OR cape verdan OR cape verdeans OR cote d'ivoire OR ivory coast OR djibouti OR egypt OR egyptian OR el salvador OR salvadoran OR fiji OR fijian OR georgia OR georgia OR ghana OR ghanaian OR guatemala OR guatemalan OR guyana OR guyanese OR honduras OR honduran OR indonesia OR indonesian OR india OR indian OR iraq OR iraqi OR kiribati OR kosovo OR kosovar OR laos OR Lao OR laotian OR lesotho OR marshall islands OR marshallese OR mauritania OR mauritanian OR micronesia OR micronesian OR moldova OR moldovan OR mongolia OR mongolian OR morocco OR moroccan OR nicaragua OR nicaraguan OR nigeria OR nigerian OR pakistan OR pakistani OR papua new guinea OR papua new guinean OR paraguay OR paraguayan OR philippines OR filipino OR samoa OR samoan OR sao tome OR principe OR santomea OR senegal OR senegalese OR solomon islands OR solomon islander OR sri lanka OR sri lankan OR sudan OR sudanese OR swazi OR swaziland OR syria OR syrian OR east timor OR east timor eye OR tonga OR tongan OR turkmenistan OR turkmen OR tuvalu OR tuvaluans OR ukraine OR ukrainian OR uzbekistan OR uzbek OR vanuatu OR vietnam OR vietnamese OR west bank OR gaza OR yemen OR yemeni OR yemenite OR zambia OR zambian OR albania OR albanian OR algeria OR algerian OR antigua and barbuda OR antigua OR barbuda OR argentina OR argentinian OR azerbaijan OR azerbaijani OR belarus OR belarusian OR bosnia OR bosnian OR botswana OR brazil OR brazilian OR bulgaria o bulgarian OR barbados OR bajan OR barbadian chile OR chilean OR china OR chinese OR colombia OR colombian OR costa rica OR costa rican OR cuba OR cuban OR dominica OR dominican OR ecuador OR ecuadorian OR gabon OR gabonese OR grenada OR grenadian OR iran OR iranian OR jamaica OR jamaican OR jordan OR jordanian OR kazakhstan OR kazakhstan OR latvia OR latvian OR lebanon OR lebanese OR libya OR libyan OR lithuania OR lithuanian OR macedonia OR macedonian OR malaysia OR malaysian OR maldives OR maldivian OR mauritius OR mauritian OR mexico OR mexican OR montenegro OR montenegrin OR namibia OR namibian OR palau OR palauan OR panama OR panamanian OR peru OR peruvian OR romania OR romanian OR russia OR russian OR serbia OR serbian OR seychelles OR seychellois OR south africa OR south african OR saint kitts OR saint lucia OR saint vincent OR suriname OR suriname OR thailand OR thai OR tunisia OR tunisian OR turkey OR turkish OR uruguay OR uruguayan OR venezuela OR venezuelan)  AND  Concept 2  (hyperlipidemias OR hyperlipidemia OR hyperlipidaemia OR hyperlipidaemias OR hyperlipemia OR hyperlipemias OR hyperlipaemia OR hyperlipaemias OR lipidemia OR high cholesterol OR hypercholesterolemia OR hypercholesterolemias OR hypercholesteremia OR hypercholesteremias OR hypercholesterolaemia OR hypercholesterolaemias OR hypercholesteraemia OR proteinuria OR Albuminuria OR Hemoglobinuria OR chronic kidney failure OR chronic kidney disease OR chronic renal disease OR chronic renal insufficiency OR CKD OR end-stage renal disease OR chronic kidney failure OR chronic kidney diseases OR chronic renal diseases OR chronic renal insufficiencies OR end-stage renal diseases OR end-stage kidney diseases OR chronic kidney failures OR chronic renal failures OR stroke OR stroke OR strokes OR brain vascular accident OR brain vascular accidents OR apoplexy OR cerebrovascular accident OR cerebrovascular accidents OR cardiomyopathies OR cardiomyopathy OR cardiomyopathies OR myocardial disease OR myocardial diseases OR myocardiopathy OR myocardiopathies OR heart neoplasms OR heart neoplasm OR cardiac tumor OR cardiac tumors OR myocardial tumor OR myocardial tumors OR cardiac carcinoma OR cardiac carcinomas OR heart cancer OR cardiac cancers OR cardiac cancer OR heart tumor OR heart tumors OR myocardial ischemia OR myocardial ischemias OR ischemic heart disease OR ischemic heart diseases OR myocardial ischaemia OR myocardial ischaemias OR ischaemic heart disease OR acute coronary syndrome OR acute coronary syndromes OR coronary disease OR coronary diseases OR coronary artery disease OR coronary artery diseases OR coronary arteriosclerosis OR Coronary atherosclerosis OR coronary stenosis OR coronary stenoses OR coronary restenosis OR coronary restenoses OR coronary heart disease OR coronary heart diseases OR coronary thrombosis OR coronary thromboses OR coronary occlusion OR coronary occlusions OR myocardial infarction OR myocardial infarctions OR heart attack OR heart attacks OR myocardial infarct OR myocardial infarcts OR heart arrest OR heart arrests OR cardiac arrest OR cardiac arrests OR asystole OR asystoles OR cardiopulmonary arrest OR cardiopulmonary arrests OR heart failure OR heart failures OR cardiac failure OR cardiac failures OR myocardial failure OR myocardial failures OR heart decompensation OR hypertension OR hypertensions OR high blood pressure OR high blood pressures OR cardiovascular diseases OR cardiovascular disease OR cardiovascular diseases OR cardiovascular risk OR cardiovascular risks)  AND  (HIV OR HIV infections OR HIV infection OR AIDS OR Human Immunodeficiency virus) |
| --- | --- |
| Intervention/Exposure | Concept 3  (Capacity building OR Organization and Administration OR Healthcare facilities, manpower and services OR Case management OR Healthcare settings OR Delivery of Health Care OR Health promotion OR Health programs OR Health Plan Implementation OR Health Systems Plans OR Public Health Systems Research OR Community Health Planning OR Program Evaluation)  AND |
| Outcome | Concept 4  Implementation outcomes: (Implementation science outcomes OR Acceptability OR Adoption OR Appropriateness OR Cost OR costs OR cost-benefit analysis OR Feasibility OR Fidelity OR Penetration OR Sustainability OR comparative effectiveness research OR delivery of health care OR delivery of healthcare OR evidence based practice OR Diffusion of Innovation OR Translational medical Research OR integration OR evidence based medicine) |

**PubMed Search strategy, indicating MeSH terms**

((((("capacity building"[MeSH Terms] OR ("capacity"[All Fields] AND "building"[All Fields]) OR "capacity building"[All Fields]) OR ("organisation and administration"[All Fields] OR "organization and administration"[Subheading] OR ("organization"[All Fields] AND "administration"[All Fields]) OR "organization and administration"[All Fields] OR "organization and administration"[MeSH Terms] OR ("organization"[All Fields] AND "administration"[All Fields])) OR (("delivery of health care"[MeSH Terms] OR ("delivery"[All Fields] AND "health"[All Fields] AND "care"[All Fields]) OR "delivery of health care"[All Fields] OR "healthcare"[All Fields]) AND facilities[All Fields] AND ("workforce"[MeSH Terms] OR "workforce"[All Fields] OR "manpower"[All Fields]) AND services[All Fields]) OR ("case management"[MeSH Terms] OR ("case"[All Fields] AND "management"[All Fields]) OR "case management"[All Fields]) OR (("delivery of health care"[MeSH Terms] OR ("delivery"[All Fields] AND "health"[All Fields] AND "care"[All Fields]) OR "delivery of health care"[All Fields] OR "healthcare"[All Fields]) AND settings[All Fields]) OR ("delivery of health care"[MeSH Terms] OR ("delivery"[All Fields] AND "health"[All Fields] AND "care"[All Fields]) OR "delivery of health care"[All Fields]) OR ("health promotion"[MeSH Terms] OR ("health"[All Fields] AND "promotion"[All Fields]) OR "health promotion"[All Fields]) OR (("health"[MeSH Terms] OR "health"[All Fields]) AND programs[All Fields]) OR ("health plan implementation"[MeSH Terms] OR ("health"[All Fields] AND "plan"[All Fields] AND "implementation"[All Fields]) OR "health plan implementation"[All Fields]) OR ("health systems plans"[MeSH Terms] OR ("health"[All Fields] AND "systems"[All Fields] AND "plans"[All Fields]) OR "health systems plans"[All Fields]) OR ("public health systems research"[MeSH Terms] OR ("public"[All Fields] AND "health"[All Fields] AND "systems"[All Fields] AND "research"[All Fields]) OR "public health systems research"[All Fields]) OR ("community health planning"[MeSH Terms] OR ("community"[All Fields] AND "health"[All Fields] AND "planning"[All Fields]) OR "community health planning"[All Fields]) OR ("programme evaluation"[All Fields] OR "program evaluation"[MeSH Terms] OR ("program"[All Fields] AND "evaluation"[All Fields]) OR "program evaluation"[All Fields])) AND ((("implementation science"[MeSH Terms] OR ("implementation"[All Fields] AND "science"[All Fields]) OR "implementation science"[All Fields]) AND outcomes[All Fields]) OR Acceptability[All Fields] OR ("adoption"[MeSH Terms] OR "adoption"[All Fields]) OR Appropriateness[All Fields] OR ("economics"[Subheading] OR "economics"[All Fields] OR "cost"[All Fields] OR "costs and cost analysis"[MeSH Terms] OR ("costs"[All Fields] AND "cost"[All Fields] AND "analysis"[All Fields]) OR "costs and cost analysis"[All Fields]) OR ("costs and cost analysis"[MeSH Terms] OR ("costs"[All Fields] AND "cost"[All Fields] AND "analysis"[All Fields]) OR "costs and cost analysis"[All Fields] OR "costs"[All Fields]) OR ("cost-benefit analysis"[MeSH Terms] OR ("cost-benefit"[All Fields] AND "analysis"[All Fields]) OR "cost-benefit analysis"[All Fields] OR ("cost"[All Fields] AND "benefit"[All Fields] AND "analysis"[All Fields]) OR "cost benefit analysis"[All Fields]) OR Feasibility[All Fields] OR Fidelity[All Fields] OR Penetration[All Fields] OR ("Sustainability"[Journal] OR "Sustainability (New Rochelle)"[Journal] OR "sustainability"[All Fields]) OR ("comparative effectiveness research"[MeSH Terms] OR ("comparative"[All Fields] AND "effectiveness"[All Fields] AND "research"[All Fields]) OR "comparative effectiveness research"[All Fields]) OR ("delivery of health care"[MeSH Terms] OR ("delivery"[All Fields] AND "health"[All Fields] AND "care"[All Fields]) OR "delivery of health care"[All Fields]) OR ("delivery of health care"[MeSH Terms] OR ("delivery"[All Fields] AND "health"[All Fields] AND "care"[All Fields]) OR "delivery of health care"[All Fields] OR ("delivery"[All Fields] AND "healthcare"[All Fields]) OR "delivery of healthcare"[All Fields]) OR ("evidence-based practice"[MeSH Terms] OR ("evidence-based"[All Fields] AND "practice"[All Fields]) OR "evidence-based practice"[All Fields] OR ("evidence"[All Fields] AND "based"[All Fields] AND "practice"[All Fields]) OR "evidence based practice"[All Fields]) OR ("diffusion of innovation"[MeSH Terms] OR ("diffusion"[All Fields] AND "innovation"[All Fields]) OR "diffusion of innovation"[All Fields]) OR ("translational medical research"[MeSH Terms] OR ("translational"[All Fields] AND "medical"[All Fields] AND "research"[All Fields]) OR "translational medical research"[All Fields]) OR ("Integration"[Journal] OR "Integration (Amst)"[Journal] OR "integration"[All Fields]) OR ("evidence-based medicine"[MeSH Terms] OR ("evidence-based"[All Fields] AND "medicine"[All Fields]) OR "evidence-based medicine"[All Fields] OR ("evidence"[All Fields] AND "based"[All Fields] AND "medicine"[All Fields]) OR "evidence based medicine"[All Fields]))) AND (("hyperlipidemias"[MeSH Terms] OR "hyperlipidemias"[All Fields]) OR ("hyperlipidaemia"[All Fields] OR "hyperlipidemias"[MeSH Terms] OR "hyperlipidemias"[All Fields] OR "hyperlipidemia"[All Fields]) OR ("hyperlipidaemia"[All Fields] OR "hyperlipidemias"[MeSH Terms] OR "hyperlipidemias"[All Fields] OR "hyperlipidemia"[All Fields]) OR ("hyperlipidemias"[MeSH Terms] OR "hyperlipidemias"[All Fields] OR "hyperlipidaemias"[All Fields]) OR ("hyperlipidemias"[MeSH Terms] OR "hyperlipidemias"[All Fields] OR "hyperlipemia"[All Fields]) OR ("hyperlipidemias"[MeSH Terms] OR "hyperlipidemias"[All Fields] OR "hyperlipemias"[All Fields]) OR ("hyperlipidemias"[MeSH Terms] OR "hyperlipidemias"[All Fields] OR "hyperlipaemia"[All Fields]) OR hyperlipaemias[All Fields] OR ("hyperlipidemias"[MeSH Terms] OR "hyperlipidemias"[All Fields] OR "lipidemia"[All Fields]) OR ("hypercholesterolemia"[MeSH Terms] OR "hypercholesterolemia"[All Fields] OR ("high"[All Fields] AND "cholesterol"[All Fields]) OR "high cholesterol"[All Fields]) OR ("hypercholesterolaemia"[All Fields] OR "hypercholesterolemia"[MeSH Terms] OR "hypercholesterolemia"[All Fields]) OR ("hypercholesterolemia"[MeSH Terms] OR "hypercholesterolemia"[All Fields] OR "hypercholesterolemias"[All Fields]) OR ("hypercholesterolemia"[MeSH Terms] OR "hypercholesterolemia"[All Fields] OR "hypercholesteremia"[All Fields]) OR ("hypercholesterolemia"[MeSH Terms] OR "hypercholesterolemia"[All Fields] OR "hypercholesteremias"[All Fields]) OR ("hypercholesterolaemia"[All Fields] OR "hypercholesterolemia"[MeSH Terms] OR "hypercholesterolemia"[All Fields]) OR hypercholesterolaemias[All Fields] OR ("hypercholesterolemia"[MeSH Terms] OR "hypercholesterolemia"[All Fields] OR "hypercholesteraemia"[All Fields]) OR ("proteinuria"[MeSH Terms] OR "proteinuria"[All Fields]) OR ("albuminuria"[MeSH Terms] OR "albuminuria"[All Fields]) OR ("haemoglobinuria"[All Fields] OR "hemoglobinuria"[MeSH Terms] OR "hemoglobinuria"[All Fields]) OR ("kidney failure, chronic"[MeSH Terms] OR ("kidney"[All Fields] AND "failure"[All Fields] AND "chronic"[All Fields]) OR "chronic kidney failure"[All Fields] OR ("chronic"[All Fields] AND "kidney"[All Fields] AND "failure"[All Fields])) OR ("renal insufficiency, chronic"[MeSH Terms] OR ("renal"[All Fields] AND "insufficiency"[All Fields] AND "chronic"[All Fields]) OR "chronic renal insufficiency"[All Fields] OR ("chronic"[All Fields] AND "kidney"[All Fields] AND "disease"[All Fields]) OR "chronic kidney disease"[All Fields]) OR ("renal insufficiency, chronic"[MeSH Terms] OR ("renal"[All Fields] AND "insufficiency"[All Fields] AND "chronic"[All Fields]) OR "chronic renal insufficiency"[All Fields] OR ("chronic"[All Fields] AND "renal"[All Fields] AND "disease"[All Fields]) OR "chronic renal disease"[All Fields] OR "kidney failure, chronic"[MeSH Terms] OR ("kidney"[All Fields] AND "failure"[All Fields] AND "chronic"[All Fields]) OR "chronic kidney failure"[All Fields] OR ("chronic"[All Fields] AND "renal"[All Fields] AND "disease"[All Fields])) OR ("renal insufficiency, chronic"[MeSH Terms] OR ("renal"[All Fields] AND "insufficiency"[All Fields] AND "chronic"[All Fields]) OR "chronic renal insufficiency"[All Fields] OR ("chronic"[All Fields] AND "renal"[All Fields] AND "insufficiency"[All Fields])) OR CKD[All Fields] OR ("kidney failure, chronic"[MeSH Terms] OR ("kidney"[All Fields] AND "failure"[All Fields] AND "chronic"[All Fields]) OR "chronic kidney failure"[All Fields] OR ("end"[All Fields] AND "stage"[All Fields] AND "renal"[All Fields] AND "disease"[All Fields]) OR "end stage renal disease"[All Fields]) OR ("kidney failure, chronic"[MeSH Terms] OR ("kidney"[All Fields] AND "failure"[All Fields] AND "chronic"[All Fields]) OR "chronic kidney failure"[All Fields] OR ("chronic"[All Fields] AND "kidney"[All Fields] AND "failure"[All Fields])) OR ("renal insufficiency, chronic"[MeSH Terms] OR ("renal"[All Fields] AND "insufficiency"[All Fields] AND "chronic"[All Fields]) OR "chronic renal insufficiency"[All Fields] OR ("chronic"[All Fields] AND "kidney"[All Fields] AND "diseases"[All Fields]) OR "chronic kidney diseases"[All Fields]) OR ("renal insufficiency, chronic"[MeSH Terms] OR ("renal"[All Fields] AND "insufficiency"[All Fields] AND "chronic"[All Fields]) OR "chronic renal insufficiency"[All Fields] OR ("chronic"[All Fields] AND "renal"[All Fields] AND "diseases"[All Fields]) OR "chronic renal diseases"[All Fields]) OR ("renal insufficiency, chronic"[MeSH Terms] OR ("renal"[All Fields] AND "insufficiency"[All Fields] AND "chronic"[All Fields]) OR "chronic renal insufficiency"[All Fields] OR ("chronic"[All Fields] AND "renal"[All Fields] AND "insufficiencies"[All Fields]) OR "chronic renal insufficiencies"[All Fields]) OR (end-stage[All Fields] AND ("kidney diseases"[MeSH Terms] OR ("kidney"[All Fields] AND "diseases"[All Fields]) OR "kidney diseases"[All Fields] OR ("renal"[All Fields] AND "diseases"[All Fields]) OR "renal diseases"[All Fields])) OR (end-stage[All Fields] AND ("kidney diseases"[MeSH Terms] OR ("kidney"[All Fields] AND "diseases"[All Fields]) OR "kidney diseases"[All Fields])) OR (chronic[All Fields] AND ("renal insufficiency"[MeSH Terms] OR ("renal"[All Fields] AND "insufficiency"[All Fields]) OR "renal insufficiency"[All Fields] OR ("kidney"[All Fields] AND "failures"[All Fields]) OR "kidney failures"[All Fields])) OR (chronic[All Fields] AND ("renal insufficiency"[MeSH Terms] OR ("renal"[All Fields] AND "insufficiency"[All Fields]) OR "renal insufficiency"[All Fields] OR ("renal"[All Fields] AND "failures"[All Fields]) OR "renal failures"[All Fields])) OR ("stroke"[MeSH Terms] OR "stroke"[All Fields]) OR ("stroke"[MeSH Terms] OR "stroke"[All Fields]) OR ("stroke"[MeSH Terms] OR "stroke"[All Fields] OR "strokes"[All Fields]) OR ("stroke"[MeSH Terms] OR "stroke"[All Fields] OR ("brain"[All Fields] AND "vascular"[All Fields] AND "accident"[All Fields]) OR "brain vascular accident"[All Fields]) OR ("stroke"[MeSH Terms] OR "stroke"[All Fields] OR ("brain"[All Fields] AND "vascular"[All Fields] AND "accidents"[All Fields]) OR "brain vascular accidents"[All Fields]) OR ("stroke"[MeSH Terms] OR "stroke"[All Fields] OR "apoplexy"[All Fields]) OR ("stroke"[MeSH Terms] OR "stroke"[All Fields] OR ("cerebrovascular"[All Fields] AND "accident"[All Fields]) OR "cerebrovascular accident"[All Fields]) OR ("stroke"[MeSH Terms] OR "stroke"[All Fields] OR ("cerebrovascular"[All Fields] AND "accidents"[All Fields]) OR "cerebrovascular accidents"[All Fields]) OR ("cardiomyopathies"[MeSH Terms] OR "cardiomyopathies"[All Fields]) OR ("cardiomyopathies"[MeSH Terms] OR "cardiomyopathies"[All Fields] OR "cardiomyopathy"[All Fields]) OR ("cardiomyopathies"[MeSH Terms] OR "cardiomyopathies"[All Fields]) OR ("cardiomyopathies"[MeSH Terms] OR "cardiomyopathies"[All Fields] OR ("myocardial"[All Fields] AND "disease"[All Fields]) OR "myocardial disease"[All Fields]) OR ("cardiomyopathies"[MeSH Terms] OR "cardiomyopathies"[All Fields] OR ("myocardial"[All Fields] AND "diseases"[All Fields]) OR "myocardial diseases"[All Fields]) OR ("cardiomyopathies"[MeSH Terms] OR "cardiomyopathies"[All Fields] OR "myocardiopathy"[All Fields]) OR ("cardiomyopathies"[MeSH Terms] OR "cardiomyopathies"[All Fields] OR "myocardiopathies"[All Fields]) OR ("heart neoplasms"[MeSH Terms] OR ("heart"[All Fields] AND "neoplasms"[All Fields]) OR "heart neoplasms"[All Fields]) OR ("heart neoplasms"[MeSH Terms] OR ("heart"[All Fields] AND "neoplasms"[All Fields]) OR "heart neoplasms"[All Fields] OR ("heart"[All Fields] AND "neoplasm"[All Fields]) OR "heart neoplasm"[All Fields]) OR ("heart neoplasms"[MeSH Terms] OR ("heart"[All Fields] AND "neoplasms"[All Fields]) OR "heart neoplasms"[All Fields] OR ("cardiac"[All Fields] AND "tumor"[All Fields]) OR "cardiac tumor"[All Fields]) OR ("heart neoplasms"[MeSH Terms] OR ("heart"[All Fields] AND "neoplasms"[All Fields]) OR "heart neoplasms"[All Fields] OR ("cardiac"[All Fields] AND "tumors"[All Fields]) OR "cardiac tumors"[All Fields]) OR (("myocardium"[MeSH Terms] OR "myocardium"[All Fields] OR "myocardial"[All Fields]) AND ("tumour"[All Fields] OR "neoplasms"[MeSH Terms] OR "neoplasms"[All Fields] OR "tumor"[All Fields])) OR (("myocardium"[MeSH Terms] OR "myocardium"[All Fields] OR "myocardial"[All Fields]) AND ("tumours"[All Fields] OR "neoplasms"[MeSH Terms] OR "neoplasms"[All Fields] OR "tumors"[All Fields])) OR ("heart neoplasms"[MeSH Terms] OR ("heart"[All Fields] AND "neoplasms"[All Fields]) OR "heart neoplasms"[All Fields] OR ("cardiac"[All Fields] AND "carcinoma"[All Fields]) OR "cardiac carcinoma"[All Fields]) OR ("heart neoplasms"[MeSH Terms] OR ("heart"[All Fields] AND "neoplasms"[All Fields]) OR "heart neoplasms"[All Fields] OR ("cardiac"[All Fields] AND "carcinomas"[All Fields]) OR "cardiac carcinomas"[All Fields]) OR ("heart neoplasms"[MeSH Terms] OR ("heart"[All Fields] AND "neoplasms"[All Fields]) OR "heart neoplasms"[All Fields] OR ("heart"[All Fields] AND "cancer"[All Fields]) OR "heart cancer"[All Fields]) OR ("heart neoplasms"[MeSH Terms] OR ("heart"[All Fields] AND "neoplasms"[All Fields]) OR "heart neoplasms"[All Fields] OR ("cardiac"[All Fields] AND "cancers"[All Fields]) OR "cardiac cancers"[All Fields]) OR ("heart neoplasms"[MeSH Terms] OR ("heart"[All Fields] AND "neoplasms"[All Fields]) OR "heart neoplasms"[All Fields] OR ("cardiac"[All Fields] AND "cancer"[All Fields]) OR "cardiac cancer"[All Fields]) OR ("heart neoplasms"[MeSH Terms] OR ("heart"[All Fields] AND "neoplasms"[All Fields]) OR "heart neoplasms"[All Fields] OR ("heart"[All Fields] AND "tumor"[All Fields]) OR "heart tumor"[All Fields]) OR ("heart neoplasms"[MeSH Terms] OR ("heart"[All Fields] AND "neoplasms"[All Fields]) OR "heart neoplasms"[All Fields] OR ("heart"[All Fields] AND "tumors"[All Fields]) OR "heart tumors"[All Fields]) OR ("myocardial ischaemia"[All Fields] OR "myocardial ischemia"[MeSH Terms] OR ("myocardial"[All Fields] AND "ischemia"[All Fields]) OR "myocardial ischemia"[All Fields] OR "coronary artery disease"[MeSH Terms] OR ("coronary"[All Fields] AND "artery"[All Fields] AND "disease"[All Fields]) OR "coronary artery disease"[All Fields] OR ("myocardial"[All Fields] AND "ischemia"[All Fields])) OR ("myocardial ischemia"[MeSH Terms] OR ("myocardial"[All Fields] AND "ischemia"[All Fields]) OR "myocardial ischemia"[All Fields] OR ("myocardial"[All Fields] AND "ischemias"[All Fields]) OR "myocardial ischemias"[All Fields]) OR ("ischaemic heart disease"[All Fields] OR "myocardial ischemia"[MeSH Terms] OR ("myocardial"[All Fields] AND "ischemia"[All Fields]) OR "myocardial ischemia"[All Fields] OR ("ischemic"[All Fields] AND "heart"[All Fields] AND "disease"[All Fields]) OR "ischemic heart disease"[All Fields] OR "coronary artery disease"[MeSH Terms] OR ("coronary"[All Fields] AND "artery"[All Fields] AND "disease"[All Fields]) OR "coronary artery disease"[All Fields] OR ("ischemic"[All Fields] AND "heart"[All Fields] AND "disease"[All Fields])) OR ("ischaemic heart diseases"[All Fields] OR "myocardial ischemia"[MeSH Terms] OR ("myocardial"[All Fields] AND "ischemia"[All Fields]) OR "myocardial ischemia"[All Fields] OR ("ischemic"[All Fields] AND "heart"[All Fields] AND "diseases"[All Fields]) OR "ischemic heart diseases"[All Fields]) OR ("myocardial ischaemia"[All Fields] OR "myocardial ischemia"[MeSH Terms] OR ("myocardial"[All Fields] AND "ischemia"[All Fields]) OR "myocardial ischemia"[All Fields] OR "coronary artery disease"[MeSH Terms] OR ("coronary"[All Fields] AND "artery"[All Fields] AND "disease"[All Fields]) OR "coronary artery disease"[All Fields] OR ("myocardial"[All Fields] AND "ischemia"[All Fields])) OR (("myocardium"[MeSH Terms] OR "myocardium"[All Fields] OR "myocardial"[All Fields]) AND ischaemias[All Fields]) OR ("ischaemic heart disease"[All Fields] OR "myocardial ischemia"[MeSH Terms] OR ("myocardial"[All Fields] AND "ischemia"[All Fields]) OR "myocardial ischemia"[All Fields] OR ("ischemic"[All Fields] AND "heart"[All Fields] AND "disease"[All Fields]) OR "ischemic heart disease"[All Fields] OR "coronary artery disease"[MeSH Terms] OR ("coronary"[All Fields] AND "artery"[All Fields] AND "disease"[All Fields]) OR "coronary artery disease"[All Fields] OR ("ischemic"[All Fields] AND "heart"[All Fields] AND "disease"[All Fields])) OR ("acute coronary syndrome"[MeSH Terms] OR ("acute"[All Fields] AND "coronary"[All Fields] AND "syndrome"[All Fields]) OR "acute coronary syndrome"[All Fields]) OR ("acute coronary syndrome"[MeSH Terms] OR ("acute"[All Fields] AND "coronary"[All Fields] AND "syndrome"[All Fields]) OR "acute coronary syndrome"[All Fields] OR ("acute"[All Fields] AND "coronary"[All Fields] AND "syndromes"[All Fields]) OR "acute coronary syndromes"[All Fields]) OR ("coronary disease"[MeSH Terms] OR ("coronary"[All Fields] AND "disease"[All Fields]) OR "coronary disease"[All Fields]) OR ("coronary disease"[MeSH Terms] OR ("coronary"[All Fields] AND "disease"[All Fields]) OR "coronary disease"[All Fields] OR ("coronary"[All Fields] AND "diseases"[All Fields]) OR "coronary diseases"[All Fields]) OR ("coronary artery disease"[MeSH Terms] OR ("coronary"[All Fields] AND "artery"[All Fields] AND "disease"[All Fields]) OR "coronary artery disease"[All Fields]) OR ("coronary artery disease"[MeSH Terms] OR ("coronary"[All Fields] AND "artery"[All Fields] AND "disease"[All Fields]) OR "coronary artery disease"[All Fields] OR ("coronary"[All Fields] AND "artery"[All Fields] AND "diseases"[All Fields]) OR "coronary artery diseases"[All Fields]) OR ("coronary artery disease"[MeSH Terms] OR ("coronary"[All Fields] AND "artery"[All Fields] AND "disease"[All Fields]) OR "coronary artery disease"[All Fields] OR ("coronary"[All Fields] AND "arteriosclerosis"[All Fields]) OR "coronary arteriosclerosis"[All Fields]) OR ("coronary artery disease"[MeSH Terms] OR ("coronary"[All Fields] AND "artery"[All Fields] AND "disease"[All Fields]) OR "coronary artery disease"[All Fields] OR ("coronary"[All Fields] AND "atherosclerosis"[All Fields]) OR "coronary atherosclerosis"[All Fields]) OR ("coronary stenosis"[MeSH Terms] OR ("coronary"[All Fields] AND "stenosis"[All Fields]) OR "coronary stenosis"[All Fields]) OR ("coronary stenosis"[MeSH Terms] OR ("coronary"[All Fields] AND "stenosis"[All Fields]) OR "coronary stenosis"[All Fields] OR ("coronary"[All Fields] AND "stenoses"[All Fields]) OR "coronary stenoses"[All Fields]) OR ("coronary restenosis"[MeSH Terms] OR ("coronary"[All Fields] AND "restenosis"[All Fields]) OR "coronary restenosis"[All Fields]) OR ("coronary restenosis"[MeSH Terms] OR ("coronary"[All Fields] AND "restenosis"[All Fields]) OR "coronary restenosis"[All Fields] OR ("coronary"[All Fields] AND "restenoses"[All Fields]) OR "coronary restenoses"[All Fields]) OR ("coronary disease"[MeSH Terms] OR ("coronary"[All Fields] AND "disease"[All Fields]) OR "coronary disease"[All Fields] OR ("coronary"[All Fields] AND "heart"[All Fields] AND "disease"[All Fields]) OR "coronary heart disease"[All Fields]) OR ("coronary disease"[MeSH Terms] OR ("coronary"[All Fields] AND "disease"[All Fields]) OR "coronary disease"[All Fields] OR ("coronary"[All Fields] AND "heart"[All Fields] AND "diseases"[All Fields]) OR "coronary heart diseases"[All Fields]) OR ("coronary thrombosis"[MeSH Terms] OR ("coronary"[All Fields] AND "thrombosis"[All Fields]) OR "coronary thrombosis"[All Fields]) OR ("coronary thrombosis"[MeSH Terms] OR ("coronary"[All Fields] AND "thrombosis"[All Fields]) OR "coronary thrombosis"[All Fields] OR ("coronary"[All Fields] AND "thromboses"[All Fields]) OR "coronary thromboses"[All Fields]) OR ("coronary occlusion"[MeSH Terms] OR ("coronary"[All Fields] AND "occlusion"[All Fields]) OR "coronary occlusion"[All Fields]) OR ("coronary occlusion"[MeSH Terms] OR ("coronary"[All Fields] AND "occlusion"[All Fields]) OR "coronary occlusion"[All Fields] OR ("coronary"[All Fields] AND "occlusions"[All Fields]) OR "coronary occlusions"[All Fields]) OR ("myocardial infarction"[MeSH Terms] OR ("myocardial"[All Fields] AND "infarction"[All Fields]) OR "myocardial infarction"[All Fields]) OR ("myocardial infarction"[MeSH Terms] OR ("myocardial"[All Fields] AND "infarction"[All Fields]) OR "myocardial infarction"[All Fields] OR ("myocardial"[All Fields] AND "infarctions"[All Fields]) OR "myocardial infarctions"[All Fields]) OR ("myocardial infarction"[MeSH Terms] OR ("myocardial"[All Fields] AND "infarction"[All Fields]) OR "myocardial infarction"[All Fields] OR ("heart"[All Fields] AND "attack"[All Fields]) OR "heart attack"[All Fields]) OR ("myocardial infarction"[MeSH Terms] OR ("myocardial"[All Fields] AND "infarction"[All Fields]) OR "myocardial infarction"[All Fields] OR ("heart"[All Fields] AND "attacks"[All Fields]) OR "heart attacks"[All Fields]) OR ("myocardial infarction"[MeSH Terms] OR ("myocardial"[All Fields] AND "infarction"[All Fields]) OR "myocardial infarction"[All Fields] OR ("myocardial"[All Fields] AND "infarct"[All Fields]) OR "myocardial infarct"[All Fields]) OR ("myocardial infarction"[MeSH Terms] OR ("myocardial"[All Fields] AND "infarction"[All Fields]) OR "myocardial infarction"[All Fields] OR ("myocardial"[All Fields] AND "infarcts"[All Fields]) OR "myocardial infarcts"[All Fields]) OR ("heart arrest"[MeSH Terms] OR ("heart"[All Fields] AND "arrest"[All Fields]) OR "heart arrest"[All Fields]) OR (("heart"[MeSH Terms] OR "heart"[All Fields]) AND arrests[All Fields]) OR ("heart arrest"[MeSH Terms] OR ("heart"[All Fields] AND "arrest"[All Fields]) OR "heart arrest"[All Fields] OR ("cardiac"[All Fields] AND "arrest"[All Fields]) OR "cardiac arrest"[All Fields]) OR (("heart"[MeSH Terms] OR "heart"[All Fields] OR "cardiac"[All Fields]) AND arrests[All Fields]) OR ("heart arrest"[MeSH Terms] OR ("heart"[All Fields] AND "arrest"[All Fields]) OR "heart arrest"[All Fields] OR "asystole"[All Fields]) OR ("heart arrest"[MeSH Terms] OR ("heart"[All Fields] AND "arrest"[All Fields]) OR "heart arrest"[All Fields] OR "asystoles"[All Fields]) OR ("heart arrest"[MeSH Terms] OR ("heart"[All Fields] AND "arrest"[All Fields]) OR "heart arrest"[All Fields] OR ("cardiopulmonary"[All Fields] AND "arrest"[All Fields]) OR "cardiopulmonary arrest"[All Fields]) OR (cardiopulmonary[All Fields] AND arrests[All Fields]) OR ("heart failure"[MeSH Terms] OR ("heart"[All Fields] AND "failure"[All Fields]) OR "heart failure"[All Fields]) OR ("heart failure"[MeSH Terms] OR ("heart"[All Fields] AND "failure"[All Fields]) OR "heart failure"[All Fields] OR ("heart"[All Fields] AND "failures"[All Fields]) OR "heart failures"[All Fields]) OR ("heart failure"[MeSH Terms] OR ("heart"[All Fields] AND "failure"[All Fields]) OR "heart failure"[All Fields] OR ("cardiac"[All Fields] AND "failure"[All Fields]) OR "cardiac failure"[All Fields]) OR ("heart failure"[MeSH Terms] OR ("heart"[All Fields] AND "failure"[All Fields]) OR "heart failure"[All Fields] OR ("cardiac"[All Fields] AND "failures"[All Fields]) OR "cardiac failures"[All Fields]) OR ("heart failure"[MeSH Terms] OR ("heart"[All Fields] AND "failure"[All Fields]) OR "heart failure"[All Fields] OR ("myocardial"[All Fields] AND "failure"[All Fields]) OR "myocardial failure"[All Fields]) OR (("myocardium"[MeSH Terms] OR "myocardium"[All Fields] OR "myocardial"[All Fields]) AND failures[All Fields]) OR ("heart failure"[MeSH Terms] OR ("heart"[All Fields] AND "failure"[All Fields]) OR "heart failure"[All Fields] OR ("heart"[All Fields] AND "decompensation"[All Fields]) OR "heart decompensation"[All Fields]) OR ("hypertension"[MeSH Terms] OR "hypertension"[All Fields]) OR hypertensions[All Fields] OR ("hypertension"[MeSH Terms] OR "hypertension"[All Fields] OR ("high"[All Fields] AND "blood"[All Fields] AND "pressure"[All Fields]) OR "high blood pressure"[All Fields]) OR ("hypertension"[MeSH Terms] OR "hypertension"[All Fields] OR ("high"[All Fields] AND "blood"[All Fields] AND "pressures"[All Fields]) OR "high blood pressures"[All Fields]) OR ("cardiovascular diseases"[MeSH Terms] OR ("cardiovascular"[All Fields] AND "diseases"[All Fields]) OR "cardiovascular diseases"[All Fields]) OR ("cardiovascular diseases"[MeSH Terms] OR ("cardiovascular"[All Fields] AND "diseases"[All Fields]) OR "cardiovascular diseases"[All Fields] OR ("cardiovascular"[All Fields] AND "disease"[All Fields]) OR "cardiovascular disease"[All Fields]) OR ("cardiovascular diseases"[MeSH Terms] OR ("cardiovascular"[All Fields] AND "diseases"[All Fields]) OR "cardiovascular diseases"[All Fields]) OR (("cardiovascular system"[MeSH Terms] OR ("cardiovascular"[All Fields] AND "system"[All Fields]) OR "cardiovascular system"[All Fields] OR "cardiovascular"[All Fields]) AND ("risk"[MeSH Terms] OR "risk"[All Fields])) OR (("cardiovascular system"[MeSH Terms] OR ("cardiovascular"[All Fields] AND "system"[All Fields]) OR "cardiovascular system"[All Fields] OR "cardiovascular"[All Fields]) AND ("risk"[MeSH Terms] OR "risk"[All Fields] OR "risks"[All Fields])))) AND (("hiv"[MeSH Terms] OR "hiv"[All Fields]) OR ("hiv infections"[MeSH Terms] OR ("hiv"[All Fields] AND "infections"[All Fields]) OR "hiv infections"[All Fields]) OR ("hiv infections"[MeSH Terms] OR ("hiv"[All Fields] AND "infections"[All Fields]) OR "hiv infections"[All Fields] OR ("hiv"[All Fields] AND "infection"[All Fields]) OR "hiv infection"[All Fields]) OR ("acquired immunodeficiency syndrome"[MeSH Terms] OR ("acquired"[All Fields] AND "immunodeficiency"[All Fields] AND "syndrome"[All Fields]) OR "acquired immunodeficiency syndrome"[All Fields] OR "aids"[All Fields]) OR ("hiv"[MeSH Terms] OR "hiv"[All Fields] OR ("human"[All Fields] AND "immunodeficiency"[All Fields] AND "virus"[All Fields]) OR "human immunodeficiency virus"[All Fields]))) AND (("developing countries"[MeSH Terms] OR ("developing"[All Fields] AND "countries"[All Fields]) OR "developing countries"[All Fields]) OR ("developing countries"[MeSH Terms] OR ("developing"[All Fields] AND "countries"[All Fields]) OR "developing countries"[All Fields] OR ("developing"[All Fields] AND "country"[All Fields]) OR "developing country"[All Fields]) OR ("medically underserved area"[MeSH Terms] OR ("medically"[All Fields] AND "underserved"[All Fields] AND "area"[All Fields]) OR "medically underserved area"[All Fields]) OR ("medically underserved area"[MeSH Terms] OR ("medically"[All Fields] AND "underserved"[All Fields] AND "area"[All Fields]) OR "medically underserved area"[All Fields] OR ("medically"[All Fields] AND "underserved"[All Fields] AND "areas"[All Fields]) OR "medically underserved areas"[All Fields]) OR lmic[All Fields] OR (("poverty"[MeSH Terms] OR "poverty"[All Fields] OR ("low"[All Fields] AND "income"[All Fields]) OR "low income"[All Fields]) AND countries[All Fields]) OR (("poverty"[MeSH Terms] OR "poverty"[All Fields] OR ("low"[All Fields] AND "income"[All Fields]) OR "low income"[All Fields]) AND country[All Fields]) OR (middle[All Fields] AND ("income"[MeSH Terms] OR "income"[All Fields]) AND countries[All Fields]) OR (middle[All Fields] AND ("income"[MeSH Terms] OR "income"[All Fields]) AND country[All Fields]) OR ("Glob Impacts"[Journal] OR "global"[All Fields]) OR (("health resources"[MeSH Terms] OR ("health"[All Fields] AND "resources"[All Fields]) OR "health resources"[All Fields] OR "resource"[All Fields]) AND ("poverty"[MeSH Terms] OR "poverty"[All Fields] OR "poor"[All Fields])) OR (low[All Fields] AND ("health resources"[MeSH Terms] OR ("health"[All Fields] AND "resources"[All Fields]) OR "health resources"[All Fields] OR "resource"[All Fields])) OR ("developing countries"[MeSH Terms] OR ("developing"[All Fields] AND "countries"[All Fields]) OR "developing countries"[All Fields] OR ("third"[All Fields] AND "world"[All Fields] AND "country"[All Fields]) OR "third world country"[All Fields]) OR ("developing countries"[MeSH Terms] OR ("developing"[All Fields] AND "countries"[All Fields]) OR "developing countries"[All Fields] OR ("third"[All Fields] AND "world"[All Fields] AND "countries"[All Fields]) OR "third world countries"[All Fields]) OR ("africa"[MeSH Terms] OR "africa"[All Fields]) OR ("asia, central"[MeSH Terms] OR ("asia"[All Fields] AND "central"[All Fields]) OR "central asia"[All Fields] OR ("central"[All Fields] AND "asia"[All Fields])) OR ("asia, western"[MeSH Terms] OR ("asia"[All Fields] AND "western"[All Fields]) OR "western asia"[All Fields] OR ("western"[All Fields] AND "asia"[All Fields])) OR ("asia, southeastern"[MeSH Terms] OR ("asia"[All Fields] AND "southeastern"[All Fields]) OR "southeastern asia"[All Fields] OR ("southeastern"[All Fields] AND "asia"[All Fields])) OR ("indian ocean islands"[MeSH Terms] OR ("indian"[All Fields] AND "ocean"[All Fields] AND "islands"[All Fields]) OR "indian ocean islands"[All Fields]) OR ("central america"[MeSH Terms] OR ("central"[All Fields] AND "america"[All Fields]) OR "central america"[All Fields]) OR ("south america"[MeSH Terms] OR ("south"[All Fields] AND "america"[All Fields]) OR "south america"[All Fields]) OR ("europe, eastern"[MeSH Terms] OR ("europe"[All Fields] AND "eastern"[All Fields]) OR "eastern europe"[All Fields] OR ("eastern"[All Fields] AND "europe"[All Fields])) OR ("transcaucasia"[MeSH Terms] OR "transcaucasia"[All Fields]) OR ("china"[MeSH Terms] OR "china"[All Fields]) OR ("korea"[MeSH Terms] OR "korea"[All Fields]) OR ("mongolia"[MeSH Terms] OR "mongolia"[All Fields]) OR ("mexico"[MeSH Terms] OR "mexico"[All Fields]) OR ("caribbean region"[MeSH Terms] OR ("caribbean"[All Fields] AND "region"[All Fields]) OR "caribbean region"[All Fields]) OR ("pacific islands"[MeSH Terms] OR ("pacific"[All Fields] AND "islands"[All Fields]) OR "pacific islands"[All Fields]) OR ("africa"[MeSH Terms] OR "africa"[All Fields]) OR ("asia, central"[MeSH Terms] OR ("asia"[All Fields] AND "central"[All Fields]) OR "central asia"[All Fields] OR ("central"[All Fields] AND "asia"[All Fields])) OR ("asia, western"[MeSH Terms] OR ("asia"[All Fields] AND "western"[All Fields]) OR "western asia"[All Fields] OR ("western"[All Fields] AND "asia"[All Fields])) OR ("asia, southeastern"[MeSH Terms] OR ("asia"[All Fields] AND "southeastern"[All Fields]) OR "southeastern asia"[All Fields] OR ("southeastern"[All Fields] AND "asia"[All Fields])) OR ("indian ocean islands"[MeSH Terms] OR ("indian"[All Fields] AND "ocean"[All Fields] AND "islands"[All Fields]) OR "indian ocean islands"[All Fields]) OR ("central america"[MeSH Terms] OR ("central"[All Fields] AND "america"[All Fields]) OR "central america"[All Fields]) OR ("south america"[MeSH Terms] OR ("south"[All Fields] AND "america"[All Fields]) OR "south america"[All Fields]) OR ("europe, eastern"[MeSH Terms] OR ("europe"[All Fields] AND "eastern"[All Fields]) OR "eastern europe"[All Fields] OR ("eastern"[All Fields] AND "europe"[All Fields])) OR ("transcaucasia"[MeSH Terms] OR "transcaucasia"[All Fields]) OR ("west indies"[MeSH Terms] OR ("west"[All Fields] AND "indies"[All Fields]) OR "west indies"[All Fields] OR "caribbean"[All Fields] OR "caribbean region"[MeSH Terms] OR ("caribbean"[All Fields] AND "region"[All Fields]) OR "caribbean region"[All Fields]) OR ("pacific islands"[MeSH Terms] OR ("pacific"[All Fields] AND "islands"[All Fields]) OR "pacific islands"[All Fields]) OR afghan[All Fields] OR afghan[All Fields] OR ("afghanistan"[MeSH Terms] OR "afghanistan"[All Fields]) OR ("bangladesh"[MeSH Terms] OR "bangladesh"[All Fields]) OR bangladeshi[All Fields] OR ("benin"[MeSH Terms] OR "benin"[All Fields]) OR beninese[All Fields] OR ("burkina faso"[MeSH Terms] OR ("burkina"[All Fields] AND "faso"[All Fields]) OR "burkina faso"[All Fields]) OR burkinabes[All Fields] OR ("burundi"[MeSH Terms] OR "burundi"[All Fields]) OR burundian[All Fields] OR ("cambodia"[MeSH Terms] OR "cambodia"[All Fields]) OR ("asian continental ancestry group"[MeSH Terms] OR ("asian"[All Fields] AND "continental"[All Fields] AND "ancestry"[All Fields] AND "group"[All Fields]) OR "asian continental ancestry group"[All Fields] OR "cambodian"[All Fields]) OR ("central african republic"[MeSH Terms] OR ("central"[All Fields] AND "african"[All Fields] AND "republic"[All Fields]) OR "central african republic"[All Fields]) OR (central[All Fields] AND ("african continental ancestry group"[MeSH Terms] OR ("african"[All Fields] AND "continental"[All Fields] AND "ancestry"[All Fields] AND "group"[All Fields]) OR "african continental ancestry group"[All Fields] OR "african"[All Fields])) OR ("chad"[MeSH Terms] OR "chad"[All Fields]) OR chadian[All Fields] OR ("comoros"[MeSH Terms] OR "comoros"[All Fields]) OR comorian[All Fields] OR ("congo"[MeSH Terms] OR "congo"[All Fields]) OR congolese[All Fields] OR ("eritrea"[MeSH Terms] OR "eritrea"[All Fields]) OR eritrean[All Fields] OR ("ethiopia"[MeSH Terms] OR "ethiopia"[All Fields]) OR ethiopian[All Fields] OR ("gambia"[MeSH Terms] OR "gambia"[All Fields]) OR gambian[All Fields] OR ("guinea"[MeSH Terms] OR "guinea"[All Fields]) OR ("guinea"[MeSH Terms] OR "guinea"[All Fields]) OR ("haiti"[MeSH Terms] OR "haiti"[All Fields]) OR haitian[All Fields] OR ("kenya"[MeSH Terms] OR "kenya"[All Fields]) OR ("kenya"[MeSH Terms] OR "kenya"[All Fields]) OR ("korea"[MeSH Terms] OR "korea"[All Fields]) OR ("asian continental ancestry group"[MeSH Terms] OR ("asian"[All Fields] AND "continental"[All Fields] AND "ancestry"[All Fields] AND "group"[All Fields]) OR "asian continental ancestry group"[All Fields] OR "korean"[All Fields]) OR kyrgyz[All Fields] OR ("kyrgyzstan"[MeSH Terms] OR "kyrgyzstan"[All Fields]) OR ("liberia"[MeSH Terms] OR "liberia"[All Fields]) OR liberian[All Fields] OR ("madagascar"[MeSH Terms] OR "madagascar"[All Fields]) OR malagasy[All Fields] OR ("malawi"[MeSH Terms] OR "malawi"[All Fields]) OR malawian[All Fields] OR ("mali"[MeSH Terms] OR "mali"[All Fields]) OR malian[All Fields] OR ("mozambique"[MeSH Terms] OR "mozambique"[All Fields]) OR mozambican[All Fields] OR ("myanmar"[MeSH Terms] OR "myanmar"[All Fields]) OR myanmarese[All Fields] OR ("asian continental ancestry group"[MeSH Terms] OR ("asian"[All Fields] AND "continental"[All Fields] AND "ancestry"[All Fields] AND "group"[All Fields]) OR "asian continental ancestry group"[All Fields] OR "burmese"[All Fields]) OR ("nepal"[MeSH Terms] OR "nepal"[All Fields]) OR nepalese[All Fields] OR ("niger"[MeSH Terms] OR "niger"[All Fields]) OR nigerian[All Fields] OR ("rwanda"[MeSH Terms] OR "rwanda"[All Fields]) OR rwandan[All Fields] OR ("sierra leone"[MeSH Terms] OR ("sierra"[All Fields] AND "leone"[All Fields]) OR "sierra leone"[All Fields]) OR (("Sierra"[Journal] OR "sierra"[All Fields]) AND leoneans[All Fields]) OR ("somalia"[MeSH Terms] OR "somalia"[All Fields]) OR ("somalia"[MeSH Terms] OR "somalia"[All Fields]) OR ("tajikistan"[MeSH Terms] OR "tajikistan"[All Fields]) OR tajik[All Fields] OR tadzhik[All Fields] OR ("tanzania"[MeSH Terms] OR "tanzania"[All Fields]) OR tanzanian[All Fields] OR ("togo"[MeSH Terms] OR "togo"[All Fields]) OR togolese[All Fields] OR ("uganda"[MeSH Terms] OR "uganda"[All Fields]) OR ugandan[All Fields] OR ("zimbabwe"[MeSH Terms] OR "zimbabwe"[All Fields]) OR zimbabwean[All Fields] OR ("angola"[MeSH Terms] OR "angola"[All Fields]) OR ("angola"[MeSH Terms] OR "angola"[All Fields]) OR ("armenia"[MeSH Terms] OR "armenia"[All Fields]) OR armenian[All Fields] OR ("belize"[MeSH Terms] OR "belize"[All Fields]) OR belizean[All Fields] OR ("bhutan"[MeSH Terms] OR "bhutan"[All Fields]) OR bhutanese[All Fields] OR ("bolivia"[MeSH Terms] OR "bolivia"[All Fields]) OR bolivian[All Fields] OR ("cameroon"[MeSH Terms] OR "cameroon"[All Fields]) OR cameroonian[All Fields] OR ("cabo verde"[MeSH Terms] OR ("cabo"[All Fields] AND "verde"[All Fields]) OR "cabo verde"[All Fields] OR ("cape"[All Fields] AND "verde"[All Fields]) OR "cape verde"[All Fields]) OR (cape[All Fields] AND verdan[All Fields]) OR (cape[All Fields] AND verdeans[All Fields]) OR ("cote d'ivoire"[MeSH Terms] OR ("cote"[All Fields] AND "d'ivoire"[All Fields]) OR "cote d'ivoire"[All Fields]) OR ("cote d'ivoire"[MeSH Terms] OR ("cote"[All Fields] AND "d'ivoire"[All Fields]) OR "cote d'ivoire"[All Fields] OR ("ivory"[All Fields] AND "coast"[All Fields]) OR "ivory coast"[All Fields]) OR ("djibouti"[MeSH Terms] OR "djibouti"[All Fields]) OR ("egypt"[MeSH Terms] OR "egypt"[All Fields]) OR egyptian[All Fields] OR ("el salvador"[MeSH Terms] OR ("el"[All Fields] AND "salvador"[All Fields]) OR "el salvador"[All Fields]) OR salvadoran[All Fields] OR ("fiji"[MeSH Terms] OR "fiji"[All Fields]) OR fijian[All Fields] OR ("georgia"[MeSH Terms] OR "georgia"[All Fields] OR "georgia (republic)"[MeSH Terms] OR ("georgia"[All Fields] AND "(republic)"[All Fields]) OR "georgia (republic)"[All Fields]) OR ("georgia"[MeSH Terms] OR "georgia"[All Fields] OR "georgia (republic)"[MeSH Terms] OR ("georgia"[All Fields] AND "(republic)"[All Fields]) OR "georgia (republic)"[All Fields]) OR ("ghana"[MeSH Terms] OR "ghana"[All Fields]) OR ghanaian[All Fields] OR ("guatemala"[MeSH Terms] OR "guatemala"[All Fields]) OR guatemalan[All Fields] OR ("guyana"[MeSH Terms] OR "guyana"[All Fields]) OR guyanese[All Fields] OR ("honduras"[MeSH Terms] OR "honduras"[All Fields]) OR honduran[All Fields] OR ("indonesia"[MeSH Terms] OR "indonesia"[All Fields]) OR indonesian[All Fields] OR ("india"[MeSH Terms] OR "india"[All Fields]) OR indian[All Fields] OR ("iraq"[MeSH Terms] OR "iraq"[All Fields]) OR iraqi[All Fields] OR ("micronesia"[MeSH Terms] OR "micronesia"[All Fields] OR "kiribati"[All Fields]) OR ("kosovo"[MeSH Terms] OR "kosovo"[All Fields]) OR kosovar[All Fields] OR ("laos"[MeSH Terms] OR "laos"[All Fields]) OR Lao[All Fields] OR laotian[All Fields] OR ("lesotho"[MeSH Terms] OR "lesotho"[All Fields]) OR ("micronesia"[MeSH Terms] OR "micronesia"[All Fields] OR ("marshall"[All Fields] AND "islands"[All Fields]) OR "marshall islands"[All Fields]) OR marshallese[All Fields] OR ("mauritania"[MeSH Terms] OR "mauritania"[All Fields]) OR mauritanian[All Fields] OR ("micronesia"[MeSH Terms] OR "micronesia"[All Fields]) OR micronesian[All Fields] OR ("moldova"[MeSH Terms] OR "moldova"[All Fields]) OR moldovan[All Fields] OR ("mongolia"[MeSH Terms] OR "mongolia"[All Fields]) OR mongolian[All Fields] OR ("morocco"[MeSH Terms] OR "morocco"[All Fields]) OR moroccan[All Fields] OR ("nicaragua"[MeSH Terms] OR "nicaragua"[All Fields]) OR nicaraguan[All Fields] OR ("nigeria"[MeSH Terms] OR "nigeria"[All Fields]) OR nigerian[All Fields] OR ("pakistan"[MeSH Terms] OR "pakistan"[All Fields]) OR pakistani[All Fields] OR ("papua new guinea"[MeSH Terms] OR ("papua"[All Fields] AND "new"[All Fields] AND "guinea"[All Fields]) OR "papua new guinea"[All Fields]) OR (papua[All Fields] AND new[All Fields] AND guinean[All Fields]) OR ("paraguay"[MeSH Terms] OR "paraguay"[All Fields]) OR paraguayan[All Fields] OR ("philippines"[MeSH Terms] OR "philippines"[All Fields]) OR filipino[All Fields] OR ("samoa"[MeSH Terms] OR "samoa"[All Fields]) OR samoan[All Fields] OR (sao[All Fields] AND tome[All Fields]) OR principe[All Fields] OR santomea[All Fields] OR ("senegal"[MeSH Terms] OR "senegal"[All Fields]) OR senegalese[All Fields] OR ("melanesia"[MeSH Terms] OR "melanesia"[All Fields] OR ("solomon"[All Fields] AND "islands"[All Fields]) OR "solomon islands"[All Fields]) OR (solomon[All Fields] AND islander[All Fields]) OR ("sri lanka"[MeSH Terms] OR ("sri"[All Fields] AND "lanka"[All Fields]) OR "sri lanka"[All Fields]) OR (sri[All Fields] AND lankan[All Fields]) OR ("sudan"[MeSH Terms] OR "sudan"[All Fields]) OR sudanese[All Fields] OR swazi[All Fields] OR ("swaziland"[MeSH Terms] OR "swaziland"[All Fields]) OR ("syria"[MeSH Terms] OR "syria"[All Fields]) OR ("mesocricetus"[MeSH Terms] OR "mesocricetus"[All Fields] OR "syrian"[All Fields]) OR ("timor-leste"[MeSH Terms] OR "timor-leste"[All Fields] OR ("east"[All Fields] AND "timor"[All Fields]) OR "east timor"[All Fields]) OR (("timor-leste"[MeSH Terms] OR "timor-leste"[All Fields] OR ("east"[All Fields] AND "timor"[All Fields]) OR "east timor"[All Fields]) AND ("eye"[MeSH Terms] OR "eye"[All Fields])) OR ("tonga"[MeSH Terms] OR "tonga"[All Fields]) OR tongan[All Fields] OR ("turkmenistan"[MeSH Terms] OR "turkmenistan"[All Fields]) OR turkmen[All Fields] OR ("micronesia"[MeSH Terms] OR "micronesia"[All Fields] OR "tuvalu"[All Fields]) OR tuvaluans[All Fields] OR ("ukraine"[MeSH Terms] OR "ukraine"[All Fields]) OR ukrainian[All Fields] OR ("uzbekistan"[MeSH Terms] OR "uzbekistan"[All Fields]) OR uzbek[All Fields] OR ("vanuatu"[MeSH Terms] OR "vanuatu"[All Fields]) OR ("vietnam"[MeSH Terms] OR "vietnam"[All Fields]) OR ("asian continental ancestry group"[MeSH Terms] OR ("asian"[All Fields] AND "continental"[All Fields] AND "ancestry"[All Fields] AND "group"[All Fields]) OR "asian continental ancestry group"[All Fields] OR "vietnamese"[All Fields]) OR ("middle east"[MeSH Terms] OR ("middle"[All Fields] AND "east"[All Fields]) OR "middle east"[All Fields] OR ("west"[All Fields] AND "bank"[All Fields]) OR "west bank"[All Fields]) OR gaza[All Fields] OR ("yemen"[MeSH Terms] OR "yemen"[All Fields]) OR yemeni[All Fields] OR yemenite[All Fields] OR ("zambia"[MeSH Terms] OR "zambia"[All Fields]) OR zambian[All Fields] OR ("albania"[MeSH Terms] OR "albania"[All Fields]) OR albanian[All Fields] OR ("algeria"[MeSH Terms] OR "algeria"[All Fields]) OR algerian[All Fields] OR ("antigua and barbuda"[MeSH Terms] OR ("antigua"[All Fields] AND "barbuda"[All Fields]) OR "antigua and barbuda"[All Fields]) OR ("antigua and barbuda"[MeSH Terms] OR ("antigua"[All Fields] AND "barbuda"[All Fields]) OR "antigua and barbuda"[All Fields] OR "antigua"[All Fields]) OR ("antigua and barbuda"[MeSH Terms] OR ("antigua"[All Fields] AND "barbuda"[All Fields]) OR "antigua and barbuda"[All Fields] OR "barbuda"[All Fields]) OR ("argentina"[MeSH Terms] OR "argentina"[All Fields]) OR argentinian[All Fields] OR ("azerbaijan"[MeSH Terms] OR "azerbaijan"[All Fields]) OR azerbaijani[All Fields] OR ("republic of belarus"[MeSH Terms] OR ("republic"[All Fields] AND "belarus"[All Fields]) OR "republic of belarus"[All Fields] OR "belarus"[All Fields]) OR belarusian[All Fields] OR ("bosnia and herzegovina"[MeSH Terms] OR ("bosnia"[All Fields] AND "herzegovina"[All Fields]) OR "bosnia and herzegovina"[All Fields] OR "bosnia"[All Fields]) OR bosnian[All Fields] OR ("botswana"[MeSH Terms] OR "botswana"[All Fields]) OR ("brazil"[MeSH Terms] OR "brazil"[All Fields]) OR brazilian[All Fields] OR (("bulgaria"[MeSH Terms] OR "bulgaria"[All Fields]) AND o[All Fields] AND bulgarian[All Fields]) OR ("barbados"[MeSH Terms] OR "barbados"[All Fields]) OR bajan[All Fields] OR (barbadian[All Fields] AND ("chile"[MeSH Terms] OR "chile"[All Fields])) OR chilean[All Fields] OR ("china"[MeSH Terms] OR "china"[All Fields]) OR ("asian continental ancestry group"[MeSH Terms] OR ("asian"[All Fields] AND "continental"[All Fields] AND "ancestry"[All Fields] AND "group"[All Fields]) OR "asian continental ancestry group"[All Fields] OR "chinese"[All Fields]) OR ("colombia"[MeSH Terms] OR "colombia"[All Fields]) OR colombian[All Fields] OR ("costa rica"[MeSH Terms] OR ("costa"[All Fields] AND "rica"[All Fields]) OR "costa rica"[All Fields]) OR (("ribs"[MeSH Terms] OR "ribs"[All Fields] OR "costa"[All Fields]) AND rican[All Fields]) OR ("cuba"[MeSH Terms] OR "cuba"[All Fields]) OR cuban[All Fields] OR ("dominica"[MeSH Terms] OR "dominica"[All Fields]) OR dominican[All Fields] OR ("ecuador"[MeSH Terms] OR "ecuador"[All Fields]) OR ecuadorian[All Fields] OR ("gabon"[MeSH Terms] OR "gabon"[All Fields]) OR gabonese[All Fields] OR ("grenada"[MeSH Terms] OR "grenada"[All Fields]) OR grenadian[All Fields] OR ("iran"[MeSH Terms] OR "iran"[All Fields]) OR iranian[All Fields] OR ("jamaica"[MeSH Terms] OR "jamaica"[All Fields]) OR jamaican[All Fields] OR ("jordan"[MeSH Terms] OR "jordan"[All Fields]) OR jordanian[All Fields] OR ("kazakhstan"[MeSH Terms] OR "kazakhstan"[All Fields]) OR ("kazakhstan"[MeSH Terms] OR "kazakhstan"[All Fields]) OR ("latvia"[MeSH Terms] OR "latvia"[All Fields]) OR latvian[All Fields] OR ("lebanon"[MeSH Terms] OR "lebanon"[All Fields]) OR lebanese[All Fields] OR ("libya"[MeSH Terms] OR "libya"[All Fields]) OR libyan[All Fields] OR ("lithuania"[MeSH Terms] OR "lithuania"[All Fields]) OR lithuanian[All Fields] OR ("macedonia (republic)"[MeSH Terms] OR ("macedonia"[All Fields] AND "(republic)"[All Fields]) OR "macedonia (republic)"[All Fields] OR "macedonia"[All Fields]) OR macedonian[All Fields] OR ("malaysia"[MeSH Terms] OR "malaysia"[All Fields]) OR malaysian[All Fields] OR ("indian ocean islands"[MeSH Terms] OR ("indian"[All Fields] AND "ocean"[All Fields] AND "islands"[All Fields]) OR "indian ocean islands"[All Fields] OR "maldives"[All Fields]) OR maldivian[All Fields] OR ("mauritius"[MeSH Terms] OR "mauritius"[All Fields]) OR mauritian[All Fields] OR ("mexico"[MeSH Terms] OR "mexico"[All Fields]) OR mexican[All Fields] OR ("montenegro"[MeSH Terms] OR "montenegro"[All Fields]) OR montenegrin[All Fields] OR ("namibia"[MeSH Terms] OR "namibia"[All Fields]) OR namibian[All Fields] OR ("palau"[MeSH Terms] OR "palau"[All Fields]) OR palauan[All Fields] OR ("panama"[MeSH Terms] OR "panama"[All Fields]) OR panamanian[All Fields] OR ("peru"[MeSH Terms] OR "peru"[All Fields]) OR peruvian[All Fields] OR ("romania"[MeSH Terms] OR "romania"[All Fields]) OR romanian[All Fields] OR ("russia"[MeSH Terms] OR "russia"[All Fields]) OR russian[All Fields] OR ("serbia"[MeSH Terms] OR "serbia"[All Fields]) OR serbian[All Fields] OR ("seychelles"[MeSH Terms] OR "seychelles"[All Fields]) OR seychellois[All Fields] OR ("south africa"[MeSH Terms] OR ("south"[All Fields] AND "africa"[All Fields]) OR "south africa"[All Fields]) OR (south[All Fields] AND ("african continental ancestry group"[MeSH Terms] OR ("african"[All Fields] AND "continental"[All Fields] AND "ancestry"[All Fields] AND "group"[All Fields]) OR "african continental ancestry group"[All Fields] OR "african"[All Fields])) OR (("saints"[MeSH Terms] OR "saints"[All Fields] OR "saint"[All Fields]) AND kitts[All Fields]) OR ("saint lucia"[MeSH Terms] OR ("saint"[All Fields] AND "lucia"[All Fields]) OR "saint lucia"[All Fields]) OR saint vincent[Author] OR ("suriname"[MeSH Terms] OR "suriname"[All Fields]) OR ("suriname"[MeSH Terms] OR "suriname"[All Fields]) OR ("thailand"[MeSH Terms] OR "thailand"[All Fields]) OR ("asian continental ancestry group"[MeSH Terms] OR ("asian"[All Fields] AND "continental"[All Fields] AND "ancestry"[All Fields] AND "group"[All Fields]) OR "asian continental ancestry group"[All Fields] OR "thai"[All Fields]) OR ("tunisia"[MeSH Terms] OR "tunisia"[All Fields]) OR tunisian[All Fields] OR ("turkey"[MeSH Terms] OR "turkey"[All Fields]) OR turkish[All Fields] OR ("uruguay"[MeSH Terms] OR "uruguay"[All Fields]) OR uruguayan[All Fields] OR ("venezuela"[MeSH Terms] OR "venezuela"[All Fields]) OR venezuelan[All Fields])

Limit to manuscripts written or translated to English. Exclude all review articles.
