# Supplementary material for: Feasibility of integrated, multilevel care for cardiovascular diseases (CVD) and HIV in low- and middle-income countries (LMICs): A scoping review
Source: PLoS One. 2019 Feb 22;14(2):e0212296. doi: 10.1371/journal.pone.0212296 (PMC6386271; doi:10.1371/journal.pone.0212296)
Supplement: S1 Text — (DOCX) [file pone.0212296.s003.docx]

**S1 Text**

**List of abbreviations**

LMICs- Low and Middle Income countries

SSA- Sub-Saharan Africa

EBIs- Evidence-based interventions

CVD- Cardiovascular disease

RCT- Randomized Control Trial

NIH- National Institute of Health

WHO- World Health Organization

GEM- Grid Enabled Measures initiative

NCD- Noncommunicable diseases

NCD-MAP- NCD Multisectoral Action Plan (MAP) tool

PRISMA- Preferred Reporting Items for Systematic Reviews and Meta-Analyses

DO- Deborah Onakomaiya

JG- Joyce Gyamfi

JI- Juliet Iwelunmor

JO- Jumoke Opeyemi

MO- Mofetoluwa Oluwasanmi

CU- Chisom Obiezu-Umeh

MD- Milena Dalton

TO- Temitope Ojo

LL- Lynette Lester

AA- Angela Aifah

SN- Shreya Nagendra

UN- Ucheoma Nwaozuru

DV- Dorice Vieira

GO- Gbenga Ogedegbe

BBA- Bernadette Boden-Albala
