# Supplementary material for: Feasibility of integrated, multilevel care for cardiovascular diseases (CVD) and HIV in low- and middle-income countries (LMICs): A scoping review
Source: PLoS One. 2019 Feb 22;14(2):e0212296. doi: 10.1371/journal.pone.0212296 (PMC6386271; doi:10.1371/journal.pone.0212296)
Supplement: S1 Protocol — (DOCX) [file pone.0212296.s004.docx]

**Feasibility of integrated, multilevel care and management for cardiovascular diseases (CVD) and HIV in low-and-middle income countries (LMICs): a scoping review protocol.**

Temitope Ojo, MPH^1¶^, Lynette Lester, B.Sc^2¶^, Juliet Iwelunmor, PhD^3¶^, Joyce Gyamfi, MS^4¶^, Chisom Obiezu-Umeh, MPH^1&^, Deborah Onakomaiya, MPH^4&^, Angela Aifah, PhD^4&^, Shreya Nagendra, MPH^3&^, Jumoke Opeyemi B.Sc^1&^, Mofetoluwa Oluwasanmi B.Sc^3&^, Milena Dalton, MPH^4&^, Ucheoma Nwaozuru, MS^3&^, Dorice Vieira, MLS, MA, MPH^1,4,5¶^, Gbenga Ogedegbe, MD^4¶*^ and Bernadette Boden-Albala, Dr.PH^1,6,7¶^.

^1^Department of Epidemiology, New York University College of Global Public Health, New York, New York, United States of America

^2^New York University School of Medicine, New York, New York, United States of America ^3^Department of Behavioral Sciences and Health Education, College for Public Health & Social Justice, Saint Louis University, Saint Louis, Missouri. United States of America

^4^Section for Global Health, Department of Population Health, New York University School of Medicine, New York, New York, United States of America

^5^New York University Health Sciences Library, New York, New York, United States of America

^6^Department of Epidemiology, New York University College of Dentistry, New York, New York, United States of America

^7^Department of Neurology, Langone School of Medicine, New York University, New York, New York, United States of America

*Corresponding author

[Olugbenga.Ogedegbe@nyumc.org](mailto:Olugbenga.Ogedegbe@nyumc.org) (GO)

^¶^These authors contributed equally to this work

^&^These authors also contributed equally to this work

**Review questions and objectives**

What is the feasibility of implementing integrated, multilevel care and management for HIV and CVD in LMICs?

The main objective of this scoping review is to synthesize evidence of feasibility of multilevel integration of cardiovascular disease (CVD) and HIV care in low and middle income countries (LMICs). More specific objectives are as follows:

- Present the concept, taxonomy, and alignment of feasibility as reported in integrated CVD-HIV care studies in LMICs with established definitions in implementation science and intervention evaluation.
- Elucidate specific metrics used by researchers to assess the feasibility of integrated CVD-HIV care interventions in LMICs as a precursor to standardizing feasibility metrics, unique to implementation climate in LMICs.

**Background**

Low and middle income countries (LMICs) are experiencing a rapid peak in chronic diseases (1). This phenomenon is steadily contributing to the growing double burden of chronic and infectious diseases in these countries. Cardiovascular diseases (CVDs), aided by related chronic conditions such as hypertension and diabetes have become the leading cause of the death globally, with LMICs experiencing about 80% of deaths from CVDs (2). Parallel to this public health challenge, is the transitioning of HIV from a fatal disease to a chronic condition amongst many people living with HIV (PLHIV). Given the aggressive global scale up of HIV care especially in LMICs, where HIV prevalence is highest, there has been a reduction in new HIV infections, with PLHIV living longer and aging (3). As a result, PLHIV are developing chronic conditions and experiencing chronic disease-related events, of which CVD and CVD-related events are most prevalent (3).

Integrating services to address CVD and HIV was inevitable and, till date, is the most practical means of tackling CVD burden amongst PLHIV. Integrated care for HIV and NCDs in this context is defined as the strategic bringing together and operationalizing of services, delivery points, technologies, modified processes and management decisions occurring at different levels of service delivery, to manage HIV and CVD risk factors for patients with or without HIV/AIDS (4).

A recent narrative review on integrated HIV-CVD care also documents the different models of integration and instances where they have been employed for the co-management of CVD and HIV (5). The integration models featured were: integrated HIV and AIDS services with CVD services, integrated CVD services with HIV and AIDS services and simultaneous integration of CVD and HIV and AIDS services (5).

According to Proctor et al. (2011), expected outcomes of interventions to integrate CVD and HIV care should be viewed in these three buckets: clinical outcomes, service outcomes and implementation outcomes (6). Implementation outcomes are precursors to how successful an intervention would be in the in the long run (6). Recent reviews of integrated CVD and HIV management in LMICs have documented a combination of mostly clinical outcomes (rise in CD4 counts, lowering of blood pressure and HbA1c) and service outcomes (improved quality of care, detection of HIV cases) but not much on implementation outcomes (acceptability, adoption, appropriateness, feasibility, costs, fidelity, penetration and sustainability) (4, 5).

**Rationale**

There is need to monitor the progress of these integrated services, given the reality of health systems challenges in LMICs, the limited resources in these countries and the undeterred growth of chronic disease burden. More importantly, monitoring and evaluation of these efforts need to be timely, and poised to capture occurring and foreseeable kinks in the process. This can be achieved by monitoring and evaluating the process of implementation for the following implementation outcomes: adoption, acceptability, appropriateness, feasibility, cost-effectiveness, penetration, fidelity and sustainability (6). To ensure the success of timely and proactive interventions to reduce CVD burden in LMICs, through an integrated, multilevel management approach, a critical analysis of early implementation stage outcomes of integrated CVD-HIV efforts is essential. Of the four early stage implementation outcomes (adoption, acceptability, appropriateness and feasibility), feasibility captures the adoption, acceptability and appropriateness of an intervention in real life settings and applications. Feasibility, as defined by Proctor and colleagues, *“is the extent to which a new treatment or innovation can be successfully used or carried out within a given agency or setting” (6).* Strategies featured in recent reviews on integrated CVD-HIV management have all been adopted and pursued to stages where their feasibility can be assessed (4, 5). As such, this scoping review on the feasibility of integrated multilevel CVD and HIV management in LMICs is necessary to:

- Present the concept, taxonomy, and alignment of feasibility as reported in integrated CVD-HIV care studies in LMICs with established definitions in implementation science and intervention evaluation.
- Elucidate specific metrics used by researchers to assess the feasibility of integrated CVD-HIV care interventions in LMICs as a precursor to standardizing feasibility metrics, unique to implementation climate in LMICs.

**Keywords:** Integrated services, cardiovascular diseases, HIV/AIDs, low and middle income countries, feasibility, health care settings, community, integration models, integration strategies, PLHIV, noncommunicable diseases, chronic diseases, hypertension, diabetes.

**Methods**

*Inclusion criteria*

We are including studies that reported on any component of an intervention used to integrate CVD management with HIV care, via quantitative, qualitative or mixed methods. According to the World Health Organization (WHO), integrated service delivery is *“the organization and management of health services so that people get the care they need, when they need it, in ways that are user-friendly, achieve the desired results and provide value for money”* (7). For the purposes of this scoping review, integrated care for HIV and NCDs is defined as the strategic bringing together and operationalizing of services, delivery points, technologies, modified processes and management decisions occurring at different levels of service delivery, to manage HIV and CVD risk factors for patients with or without HIV/AIDS (4). Integrated HIV and CVD care would be at facilities that originally offered only HIV services (Model 1), or only CVD services (Model 2), or neither HIV or CVD services (Model 3) (5), regardless of the type of care delivery setting (e.g. HIV clinics, primary care practices or community-based settings) (4, 5). We will focus on the most common CVD risk factors in patients with HIV and these include diabetes, hypertension, dyslipidemia (all of which are associated with metabolic syndrome - a common side effect of anti-retroviral drugs) (8). Similarly, stroke will be included given its high association with hypertension (9). We will include studies that met the aforementioned criteria regardless of study design or whether or not the authors reported outcome measures. Studies were restricted to those reported in or translated to English. There will be no date restrictions for the search. Included studies will be restricted to those conducted in LMICs. We defined LMICs based on the World Bank classification of countries with Gross National Income per capita less than $995 to $12,055 in 2017 (10).

During the full-text review, feasibility will be ascertained in selected articles if one or more of the following criteria are met: 1) a specific indication that a feasibility study was conducted; 2) descriptions of interventions with adjectives such as ‘feasible’ or terms synonymous with feasibility (actual fit or utility, suitability for everyday use and practicability); (6, 11) and 3) reporting one or more of the following early-stage implementation outcomes: acceptability, appropriateness, and adoption that contributes to the feasibility of EBIs (6).

*Exclusion criteria*

We will exclude systematic reviews and literature reviews. We will exclude studies and reports that presented plans and recommendations for integrating CVD and HIV care without reporting on the actual implementation of these recommendations.

*Information sources*

We will search in the following databases: PubMed/Medline, Global Health, PubMed Central (PMC), Embase, Web of Science, Scientific Electronic Library Online (SCIELO), Food Science and Technology Abstracts (FSTA), Information Services for Physics, Engineering and Computing (INSPEC). We will search grey literature searches in Google Scholar, ResearchGate, the New York Academy of Medicine (New York AM) Grey Literature database, and recently published systematic reviews on integrated chronic disease care.

*Search strategy*

The PRISMA criteria for reporting systematic reviews and meta-analysis will guide the search for, identification of, data extraction and data synthesis from selected studies. An information specialist will consult with the research team on the search strategy design for different databases and sources. Databases will be searched from their date of inception to April, 2018. Grey literature searches will be concluded by mid-May, 2018. The search strategy will include the following terms and medical subject headings: healthcare settings, implementation science outcomes, hypertension, HIV infections and LMICs. The Boolean logic strategy using a variation of keywords with the (AND/OR logic) will be applied across all the databases.

*Study selection*

Two authors (TO and LL) will separately review and assess articles by article title or title and abstract, to determine articles meet the inclusion criteria. A full-text review will be carried out to confirm that all articles selected meet the inclusion criteria. Ambiguous abstracts will also be evaluated via a full text review for eligibility. A third reviewer (SN) will resolve disagreements between reviewers on an article’s eligibility. (See **S3 Text** for full names of reviewers)

*Risk of bias assessment*

The two reviewers will assess risk of bias independently. The Cochrane Risk of Bias Tool will be used to assess risk of bias for RCTs (12). This tool assesses each item of bias either as low, high or unclear risk of bias. The Newcastle-Ottawa Quality Assessment Scale will be used to assess risk of selection bias, information bias and bias of confounding in non-RCT, observational studies (13). Each item in this tool has multiple options, the lowest risk of bias being the option(s) with a star. Based on the guidance provided for determining risk of bias by both assessment tools, we will assess risk of bias in these three categories for all the studies: low risk of bias, high risk of bias and unclear risk of bias. Low risk of bias indicates that the item on the risk of bias assessment tool was described and well accounted for in the study, using the tool’s specifications for determination. High risk of bias indicates the item of bias was not sufficiently described and tackled in the study. Unclear risk of bias indicates that there was no information provided in the studies to determine if the specific item of bias was addressed in the studies.

*Data collection process*

The two reviewers (TO and LL) will use a standardized Google form to extract study characteristics and results from the full text article review. TO and LL will resolve data discrepancies by consensus or by a third reviewer (SN). Final articles will be chosen by consensus.

*Data items and synthesis*

We will collect the following data items from the selected studies: location of intervention/programming, duration of intervention or programming, type of healthcare setting, number of facilities/sites receiving the intervention or programming, if the location was rural, urban, peri-urban or both, the model of integration used, the target recipients of the intervention or programming, the types of staff used, if the staff were new, existing or a hybrid of both, context on reported feasibility of intervention, clinical outcomes, implementation outcomes and reviewers’ (TO and LL) notes from each article. Due to the heterogeneity in the types of integrated care interventions, study design and reported outcomes, we will conduct a qualitative synthesis of the scoping review, reporting data on feasibility as observed in selected studies.

We will report selected studies by the: i) level of integration ii) stage of intervention development, iii) entry-point of intervention along the chronic disease continuum. The three levels of integration were: micro-, meso-, and macro-levels of integrations (14). Micro-level integration comprises of integrated CVD-HIV care interventions that are individual-level and patient-focused (14). An example of micro-level integration of care would be coordinated care between professionals in charge of care for an individual patient, to ensure there is no break in communication and care continuum in the individual’s experience with the health system (14). Meso-level integration represents delivery of integrated CVD-HIV care to a specific group of people with similar disease conditions (14). An example of meso-level integration of care would be integrated care for the elderly population or people with specific, long-term conditions, as these populations have a higher use of care services that are most optimal when coordinated (14). Macro-level integration represents the delivery of integrated CVD-HIV care on a larger, systems-level scale to a broader catchment of the population (14). An example of a macro-level integration of care is Kaiser Permanente, one of the largest non-profit health maintenance organizations in the United States, which integrates health plan, providers who provide outpatient care, and hospitals or facilities that deliver inpatient care. Kaiser provides care for about 12.2 million people (14, 15). At any of these levels, the integration could be clinical (shared guidelines and protocols for several clinical care processes) or service-oriented (multidisciplinary delivery of clinical services) (14). Clinical integration involves fusing care provided by different providers and professionals for patients into a single process, navigated with use of shared guidelines and protocols (14). Service integration occurs when different clinical services provided are integrated at an organizational level (hospital level or care-group level), aided with the use of multidisciplinary professional teams (14).

We will adopt the chronic disease continuum developed in the North West Adelaide Health Study, a large population-based cohort study investigating NCDs prevalence and related risk factors along the continuum (16). In this study, stages of chronic conditions were classified into those at risk of NCDs, those with a previously undiagnosed NCD, and those previously diagnosed with an NCD. The corresponding type of action for each of these stages in sequential order is: i) prevention, ii) delay/early detection, iii) prevention/ delay/early detection/ care (16).

We will use the Medical Research Council recommended stages of intervention development: intervention design/development stage, the evaluation and implementation stage, and the intervention scale-up stage (11).

**References**

1. WHO. World Health Organization. (2017a). Media sheet: Noncommunicable diseases.<http://www.who.int/mediacentre/factsheets/fs355/en/>. Updated June, 2017. Accessed December 15, 2017.
2. WHO. World Health Organization (2017b). Media Center. Cardiovascular diseases (CVD).<http://www.who.int/mediacentre/factsheets/fs317/en/>. Updated May 2017. Accessed December 15, 2017.
3. UNAIDS. (2011). Chronic care for HIV and noncommunicable diseases: How to leverage the HIV experience. Geneva: UNAIDS.
4. Haldane V, Legido-Quigley H, Chuah FLH, Sigfrid L, Murphy G, Ong SE, et al. Integrating cardiovascular diseases, hypertension, and diabetes with HIV services: a systematic review. Aids Care-Psychological and Socio-Medical Aspects of Aids/Hiv. 2018;30(1):103-15.
5. Duffy M, Ojikutu B, Andrian S, Sohng E, Minior T, Hirschhorn LR. Non‐communicable diseases and Hiv care and treatment: models of integrated service delivery. Tropical Medicine & International Health. 2017.
6. Proctor E, Silmere H, Raghavan R, Hovmand P, Aarons G, Bunger A, et al. Outcomes for implementation research: conceptual distinctions, measurement challenges, and research agenda. Administration and Policy in Mental Health and Mental Health Services Research. 2011;38(2):65-76.
7. World Health Organization W. Integrated Health Services – What and Why? Geneva; 2008.
8. Nsagha DS, Assob JCN, Njunda AL, Tanue EA, Kibu OD, Ayima CW, et al. Risk factors of cardiovascular diseases in HIV/AIDS patients on HAART. The open AIDS journal. 2015;9:51.
9. Nielsen WB, Lindenstrøm E, Vestbo J, Jensen GB. Is diastolic hypertension an independent risk factor for stroke in the presence of normal systolic blood pressure in the middle-aged and elderly? American journal of hypertension. 1997;10(6):634-9.
10. Group TWB. World Bank Country and Lending Groups. 2017 [Available from: <https://datahelpdesk.worldbank.org/knowledgebase/articles/906519-world-bank-country-and-lending-groups>.
11. Craig P, Dieppe P, Macintyre S, Michie S, Nazareth I, Petticrew M. Developing and evaluating complex interventions: the new Medical Research Council guidance. BMJ (Clinical research ed). 2008;337:a1655.
12. Collaboration TC. The Cochrane's Collaboration tool for assessing risk of bias: Cochrane Handbook for Systematic Reviews of Interventions. 2011 [Version 5.1.0:[Available from: <http://handbook-5-1.cochrane.org/chapter_8/table_8_5_a_the_cochrane_collaborations_tool_for_assessing.htm>.
13. GA Wells BS, D O'Connell, J Peterson, V Welch, M Losos, P Tugwell. The Newcastle-Ottawa Scale (NOS) for assessing the quality of nonrandomised studies in meta-analyses 2009 [Available from: <http://www.ohri.ca/programs/clinical_epidemiology/oxford.asp>.
14. Curry N, Ham C. Clinical and service integration: the route to improved outcomes: King's Fund London; 2010.
15. Permanente K. Fast facts about Kaiser Permanente 2018 [Available from: <https://share.kaiserpermanente.org/about-us/fast-facts/>.
16. Grant JF, Chittleborough CR, Taylor AW, Dal Grande E, Wilson DH, Phillips PJ, et al. The North West Adelaide Health Study: detailed methods and baseline segmentation of a cohort for selected chronic diseases. Epidemiologic Perspectives & Innovations. 2006;3(1):4.
